# Association of salivary proteins with periodontal disease in children and adolescents- a scoping review

**DOI:** 10.1186/s12903-026-08005-2

**Published:** 2026-02-28

**Authors:** Mahwish Raja, Hani Nazzal, Farhan Sachal Cyprian, Manal Matoug-Elwerfelli, Monty Duggal

**Affiliations:** 1 College of Dental Medicine, Qatar University, Doha, Qatar; 2 Paediatric Dentistry, Hamad Medical Corporation, Doha, Qatar; 3 College of Medicine, Qatar University, Doha, Qatar

**Keywords:** Adolescents, Children, Periodontal disease; Salivary proteins

## Abstract

**Purpose:**

The value of salivary proteins as diagnostic biomarkers has been investigated for a wide range of oral and systemic diseases. The purpose of this study is to review the current research evidence on the association between salivary protein profile and periodontal disease in children and adolescents between 6 and 18 years.

**Methods:**

This scoping review was informed by the Joanna Briggs Institute methodology for scoping reviews and followed the Preferred Reporting Items for Systematic reviews and Meta-Analyses extension for Scoping Reviews. Electronic searches were conducted using PubMed, Scopus, Embase, Web of Science and Google Scholar along with grey literature. The searches were limited to studies on humans, published from inception up to February 2025.The eligibility criteria were observational studies exploring correlations between salivary proteins and periodontal disease in children and adolescents during mixed and early permanent dentition (6–18 years). A descriptive analysis of the extracted data was conducted.

**Results:**

A total of 15 primary studies met the eligibility criteria. The studies included in this review were published between 2004 and 2024 and comprised 10 analytical cross-sectional studies, followed by 3 case-control studies and two cohort studies. Across the 15 studies, a total of 1610 participants were recruited which included 860 males, and 750 females. Among the various proteins investigated, inflammatory cytokines (e.g., IL-6, IL-1β, TNF-α, MIP-1α, and MMP7) consistently demonstrated a strong association with both gingival inflammation and periodontal tissue destruction. Conversely, the total protein content and antimicrobial peptides were reported by one study each, but the findings were not statistically significant.

**Conclusion:**

The synthesis of studies included in this review shows a complex, albeit preliminary association between salivary protein profiles and periodontal status in young populations. The interpretations are tempered by significant heterogeneity and methodological limitations across the included studies. Observed trends, such as the elevation of specific markers (MIP-1α, IL-6, MMP7) in disease and glycoproteins in health, must be interpreted with caution due to variations in study design, sample collection, and analytical techniques. The inconsistent findings for total protein and cystatin C further underscore the preliminary nature of these results. Therefore, while salivary proteins represent a promising avenue for future research, their role as reliable biomarkers for diagnosis and monitoring cannot be confirmed from the existing body of evidence, which highlights a need for more standardized investigations with longitudinal data.

**Scoping review registration:**

The study protocol was registered in the Open science framework (Registration DOI 10.17605/OSF.IO/WRSN4).

**Trial registration:**

Not applicable.

**Supplementary Information:**

The online version contains supplementary material available at 10.1186/s12903-026-08005-2.

## Introduction

A growing research focus has been witnessed in exploring the utility of saliva as a diagnostic fluid for oral and systemic diseases. Saliva is a biological fluid and contains a wide range of proteins, enzymes, hormones, and antibodies which may show variations during health and disease. The oral proteomic environment, specifically the type and concentration of salivary ions, proteins and polypeptide molecules are known to play a major role in maintaining the mouth in a state of health and most importantly provide a window into the health of the individual [20, 24, 58]. The comprehensive biochemical blueprint provided by saliva offers promise for early disease detection and monitoring. Saliva collection is non-invasive, simple, and cost-effective, making it suitable for routine screening and large-scale studies [29, 32].

The value of salivary proteins as diagnostic biomarkers has been investigated for a wide range of diseases including Alzheimer’s disease [40], Parkinsonism [41], cancer [16, 38, 57, 61], and mental health disorders [11, 23]. Amongst oral diseases, differential expression of salivary proteins has been associated with a number of oral pathological conditions including Sjogren’s syndrome [32], oral squamous cell carcinoma [26, 44, 60], and dental caries [3, 10, 31, 39, 48, 53].

Periodontal disease is an inflammatory condition, and several types of salivary cytokines, enzymes and other proteins have been investigated as potential biomarkers for periodontal disease. Previous studies have shown that specific cytokines are elevated in the saliva of patients with periodontal disease [42]. Inflammation in periodontal tissues leads to the release of matrix metalloproteinase (MMPs) and aspartate aminotransferase which cause collagen breakdown. With further progression of periodontal disease, the levels of TNF-α, IL-1β, IL-6 and receptor activator of nuclear factor kappa-Β ligand (RANKL) increase, leading to the resorption of alveolar bone [34].

Osteoclastic activity during periodontal inflammation may lead to bone destruction and may be associated with raised levels of Interleukins (IL) such as IL-1 and IL-6 [51]. Salivary IL-1 was found to be a significant biomarker for periodontitis and it could be used in the diagnosis of periodontal disease [25, 28]. Matrix metalloproteinases (MMPs) are a group of enzymes that play a role in immune response to periodontal inflammation and associated tissue breakdown. However, the expression of inductive MMPs can be up- or down‐regulated by pro‐ and anti‐inflammatory cytokines. MMP‐3, ‐8 and ‐9 gene protein expression by gingival fibroblasts and MMP‐13 expression by osteoblasts can be upregulated by IL‐1β and TNF‐ α [59]. MMPs have been implicated in the pathogenesis of systemic conditions due to their role in inflammation and tissue destruction. Salivary MMP-8 levels are reported to be associated with attachment loss in periodontitis. Moreover, association is also reported between salivary levels of IL-1β, MMP-8 and severe periodontitis [33, 50].

Overall, these studies suggest that salivary cytokine expression may be associated with the pathogenesis of periodontal disease and may potentially be used as biomarkers for its early detection and monitoring. Given that periodontal disease is more prevalent in mature adults, the bulk of published studies have focused on association of salivary proteins with periodontitis in adults [5, 12, 15, 55]. Recent systematic reviews have reported that some salivary proteins are consistently under-expressed or over-expressed in adults with periodontitis [5, 12]. Similarly, another systematic review also supports the use of salivary protein biomarkers to monitor periodontitis in adults [55]. Finally, a systematic review and meta-analysis reported that salivary MMP-8 may be used to differentiate between periodontal health and disease [15].

Evidence from published literature shows that cytokine expression may vary with age and may be different in children and adults [4, 27, 30]. The expression and composition of salivary proteins are known to vary across developmental stages, influenced by physiological, hormonal, and immunological changes from childhood through adolescence to adulthood [64]. These age-related variations may affect both the diagnostic reliability and the biological interpretation of proteomic markers identified in adult populations. The rationale of the current scoping review is justified as a first step to capture the landscape of research on salivary proteins and risk of periodontal disease specifically in children and adolescents, to identify knowledge gaps, and to inform future age-specific protein biomarker research and clinical applications. The aim of this scoping review was to systematically map and summarize the current literature on the association between salivary proteins and periodontal disease in children and adolescents during mixed and early permanent dentition to identify potential gaps and inform future research.

## Methods

### Study protocol and registration

This scoping review was informed by the Joanna Briggs Institute methodology for scoping reviews and followed the Preferred Reporting Items for Systematic reviews and Meta-Analyses extension for Scoping Reviews (PRISMA-ScR) [45, 62].

A PRISMA-ScR checklist is included in the supplementary data file (Table Is).

The study protocol was registered in the Open science framework (Registration DOI 10.17605/OSF.IO/WRSN4*).*

### Research question

Which salivary protein biomarkers have been studied to explore the association with periodontal disease in children and adolescents?

A population-concept-context (PCC) framework was used to answer the research question. (M. D. J. Peters, [45].The components of the PCC framework were as follows:

- *Population:* Children in the mixed dentition and adolescents (6-18 years)
- *Concept:* Salivary protein biomarkers
- *Context:* Periodontal disease in relation to salivary proteins

### Eligibility criteria

The eligibility criteria were guided by the PCC strategy and are summarized in Table 1.

**Table 1:** Eligibility Criteria

PCC Element	Inclusion Criteria	Exclusion Criteria
Population Children in the mixed dentition and adolescents (6–18 years)	Participants (6–18 years) in good medical health	• Participants with known systemic disease including severe disabilities and use of regular prescribed medications. • Severe developmental anomalies of the head and neck region
Concept (C) Salivary protein biomarkers	Studies investigating salivary proteins in relation to periodontal disease	Studies not addressing salivary proteins with periodontal disease
Context (C) Periodontal disease in relation to salivary proteins	• Observational studies *(*cohort, case-control, and analytical cross-sectional design) • Studies done on human subjects • Studies published in English • Studies published from inception to February 2025	Animal or in vitro studies, reviews, editorials, commentaries, and non-English publications

### Study selection

Observational studies *(*cohort, case-control, and analytical cross-sectional design) on humans were included in the review. Studies conducted on animal, in vitro studies, reviews, editorials, commentaries, and non-English articles were excluded as shown in Table 1.

### Information sources and search strategy

Electronic searches were conducted on PubMed, Scopus, Embase, Web of Science, and Google scholar from inception up to February 2025. A supplementary search in the grey literature was undertaken using Open Grey (https://www.opengrey.eu). Additionally, the citations in retrieved full-text articles were reviewed to identify additional studies.

The search strategy was used with appropriate syntax for individual databases. Search strings were created by using a combination of key words and index terms by integrating Boolean operators.

The following search strategy was used in PubMed and adapted for other databases.

((((((((((((((((((((((((((“saliva proteins”) OR (“saliva peptides”)) OR (“saliva proteome”)) OR (“Salivary Proteins and Peptides“[Mesh])) OR (“salivary protein biomarkers”)) OR (“salivary proteomic profile”)) OR (“salivary protein expression”)) OR (“total salivary proteins”)) OR (“Mucin-5B“[Mesh])) OR (“Salivary Proline-Rich Proteins“[Mesh])) OR (“Salivary alpha-Amylases“[Mesh])) OR (“Histatins“[Mesh])) OR (“Salivary Cystatins“[Mesh])) OR (“Matrix Metalloproteinase 8“[Mesh])) OR (“Fibronectins“[Mesh])) OR (“Antimicrobial Cationic Peptides“[Mesh])) OR (“salivary mucins”)) OR (“salivary IgA”)) OR (“salivary statherin”)) OR (salivary defensins)) OR (salivary cathelicidins)) OR (salivary human lysozyme)) OR (“salivary lactoferrin”)) OR (“salivary glycoproteins”)) OR (salivary cytokines)) OR (salivary peroxidase)) AND (((((((((“Periodontitis“[Mesh]) OR (“Gingivitis“[Mesh])) OR (“Periodontal Pocket“[Mesh])) OR (“periodontal disease”)) OR (“gingival disease”)) OR (“periodontal inflammation”)) OR (“gingival inflammation”)) OR (“gum disease”))).

Details of search terms used for individual databases are provided in the supplementary data file (Table IIs). The searches were run on 12 February 2025.

### Selection of sources of evidence

All records from initial searches were imported into reference management software EndNote^®^, version X20; (Clarivate Analytics) and duplicates were removed. Two investigators (MR and MME) carried out title and abstract screening of the studies independently. Rayyan Systematic Review Screening Software (https://www.rayyan.ai) was used to complete the screening as per the eligibility criteria. Full texts of potentially eligible studies were retrieved and evaluated independently by the same investigators. Any disagreements in screening were discussed and resolved by a third reviewer (HN). Articles that did not meet any one or more of the inclusion criteria were excluded.

### Data charting process and data items

Data extraction was performed by two independent investigators (MR and MME) and comparisons were done to evaluate accuracy of data. Any disagreement was resolved through discussion between the two reviewers. The key data extracted from selected literature included: a) study Information (author, year and country of publication); (b) study design; (c) age of subjects; (d) sample size; (e) study groups; (f) gender; (g) diagnostic criteria; (h) saliva sample; (I ) salivary proteins quantification method; (j) type of salivary proteins analysed; (k) objective; (l) main findings; (m) conclusion. The data were recorded in a standardised Microsoft Excel sheet. Emails were sent to the corresponding authors of studies if data were missing or reported inadequately. However, only published data in the included studies were used as no responses were received.

### Data synthesis

Tables were used to synthesize data extracted from the studies including research design, participants, group characteristics, diagnostic criteria, saliva sample, salivary proteins assessment methods, and types of salivary proteins assessed. Visual mapping of salivary protein quantification methods were demonstrated as a pie chart and salivary proteins identified in the included studies were depicted using a heat map. A narrative synthesis was conducted to summarize findings qualitatively in a descriptive manner for all the included studies.

### Ethics approval

Ethics approval was not required for this scoping review as it did not generate any new data. 

## Results

### Study literature search and selection

**Fig. 1:**
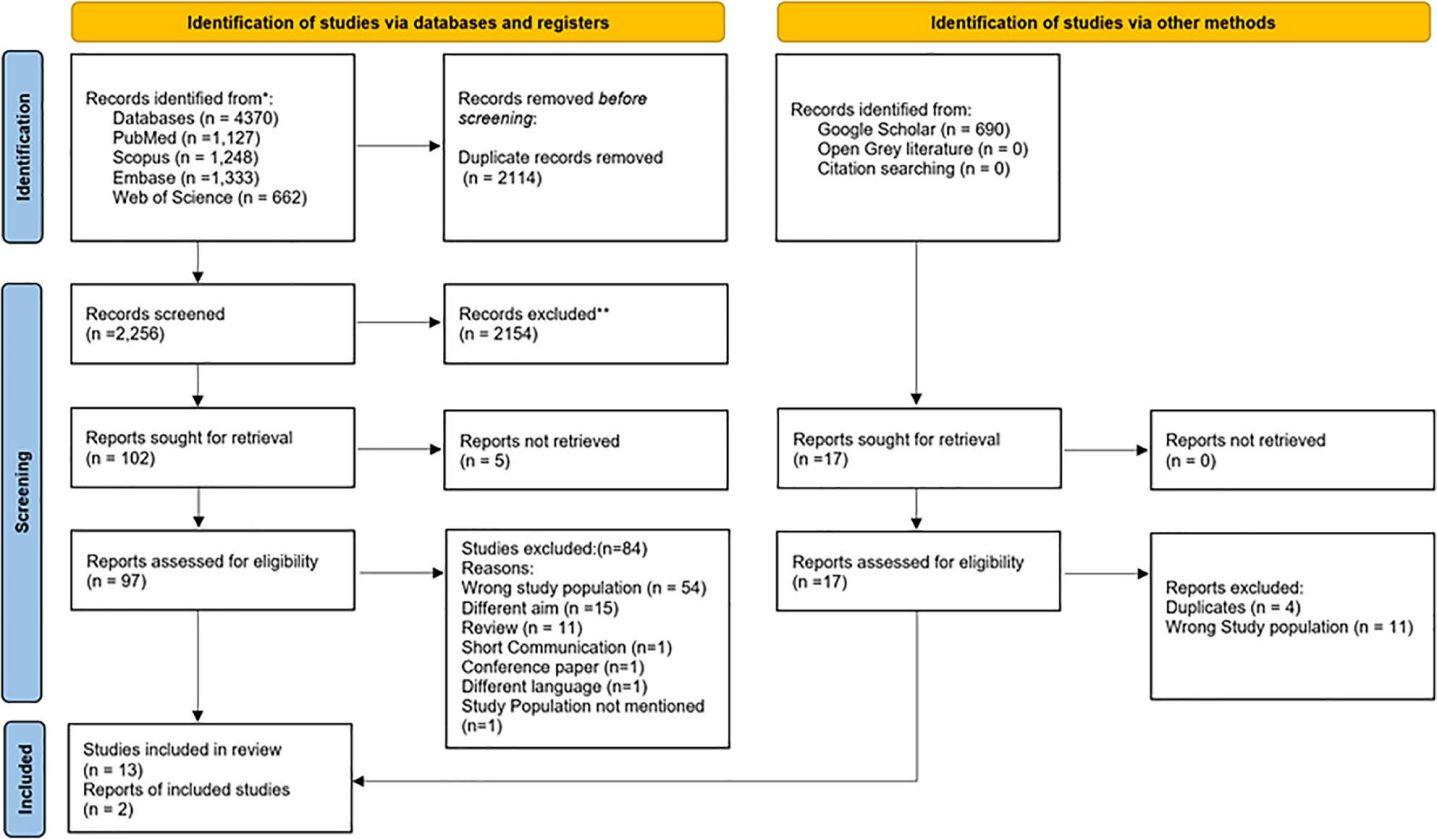
PRISMA 2020 flow diagram for scoping reviews which included searches of databases, and other sources. [62]

The initial searches on four electronic databases identified 4370 studies. After duplicate removal (*n* = 2114), this number was reduced to 2,256. Title and abstract screening showed that 2,154 studies did not meet the eligibility criteria and were excluded, and the remaining 102 articles were identified for full-text screening. Five articles could not be retrieved. Through a meticulous full text screening process, a total of 97 studies were assessed. Full text screening identified 84 articles which did not meet the eligibility criteria and were excluded. Finally, 13 studies were identified for inclusion from PubMed, Scopus, Embase and Web of Science. A parallel search on Google Scholar identified 690 studies of which 17 were assessed for eligibility. Following exclusion of 15 studies, 2 were found to be eligible for inclusion in the review after full text screening. The combined search, screening, and selection process of studies from PubMed, Scopus, Embase, Web of science and Google Scholar identified 15 primary studies for inclusion in the review as depicted in the PRISMA Flow chart (Fig. 1). No additional records were retrieved from open grey literature. The list of excluded studies along with reason of exclusion is summarized in the supplementary data file (Table IIIs).

### Primary characteristics of included studies

The studies included in this review were published between 2004 and 2024 and included 10 (66.66%) cross-sectional studies, followed by 3 (20%) case control studies and two (13.33%) cohort studies. The main characteristics of the included studies in the qualitative synthesis are summarised in Table 2.

**Table 2:** Primary characteristics of the included studies

Author / Country	Study design	Age (Years)	Sample size (*n*) & groups	Gender (Male\Female)	Diagnostic criteria	Saliva sample	Salivary proteins quantification method (s)	Salivary proteins assessed
Bimstein et al., [6] USA	Analytical cross-sectional	9–10	*30 participants* Study group (Periodontitis) (*n* = 13) Control group (Healthy) (*n* = 17)	15 males 15 females	GI PI RBL	Unstimulated saliva Collection time NR	Dip stick protein analyses	Leukocyte esterase Salivary proteins
Blufstein, [7] Austria	Analytical cross-sectional	7–12	*46 participants* Study group (Gingivitis) (*n* = 24) Control group (Healthy) (*n* = 22)	28 males 18 females	CPITN	Unstimulated saliva (8:00–11:00)	ELISA	MRP-8/14
Casarin RCV, et al., [8] Brazil	Case-control	6–12	*33 participants* Study group (Periodontitis) (*n* = 17) Control group (Healthy) (*n* = 16)	17 males 16 females	PI GI PPD CAL BOP	Unstimulated saliva Collection time NR	Bradford method LC-MS/MS	Total salivary proteins Differentially expressed salivary proteins
Chattopadhyay S et al., [9] India	Analytical cross-sectional	10–15	*79 participants* Study group (Chronic periodontitis) (*n* = 40) Control group (Healthy) (*n* = 39)	34 males 45 females	BOP PPD RBL	Stimulated saliva Collection time NR	ELISA	Total salivary proteins MUC4 MMP7
Davidovich e, et al., [13] Israel	Analytical cross-sectional	4–12	*100 participants* Divided into three age groups: 4–6 (*n* = 28) 6–11 (*n* = 62) 11–13 (*n* = 10)	51 males 49 females	PI GI	Unstimulated saliva (08:00–12:30)	Bradford method ELISA	Total salivary proteins Inflammatory cytokines: (IL-6, IL-8, IL-10 & TNFα)
Deng et al., [14] China	Analytical cross-sectional	12–15	*100 participants* Study group (More Extensive periodontitis) (*n* = 50) Control group (Less extensive periodontitis) (*n* = 50)	69 males 31 females	CPI VPI	Unstimulated saliva (09:00–12:00)	ELISA	sPLA2-IIA IL-6
Fine DH et al., [18] USA	Longitudinal cohort	11–16	*100 participants* 50 *Aggregatibacter actinomycetemcomitans* positive 50 *Aggregatibacter actinomycetemcomitans* Negative	50 males 50 females	PPD CAL RBL	5 mL Unstimulated saliva Collection time NR	Luminex xMap system	Total salivary proteins Salivary cytokines: (MIP-1α, MIP-1β, IL-α, IL-1 β & IL-8)
Fine DH et al., [17] USA	Longitudinal cohort	11–17	*48 participants* Divided into subsets based on longitudinal clinical data and microbiology results for *Aggregatibacter actinomycetemcomitans*	22 males 26 females	PPD CAL RBL	5mL Unstimulated saliva Collection time NR	Luminex xMap system	Inflammatory cytokines
Heikkinen AM et al., [22] Finland	Analytical cross-sectional	15–16	*252 participants* Grouped on the basis of periodontal pocket depth 0 Deep pockets (*n* = 73) 1 to 3 Deep pockets (*n* = 62) 4 to 7 Deep pockets (*n* = 67) > 7 Deep pockets (*n* = 50)	141 males 111 females	VPI BOP PPD CAL	Stimulated saliva Collection time NR	NR	Total salivary proteins Albumin IgA, IgG, & IgM aMMP-8 PMN elastase
Moghadam MM et al., [35] Iran	Case-control	6–7	*32 participants* Study group (Gingivitis) (*n* = 16) Control group (Healthy) (*n* = 16)	32 males	BOP GI	5mL Unstimulated saliva (10:00–12:00)	ELISA	Hydroxyproline
Monteiro MF et al., [36] Brazil	Case-control	6–12	*30 participants* Study group (Generalized Aggressive Periodontitis) (*n* = 15) Control group (Healthy) (*n* = 15)	18 males 12 females	PI GI BOP PPD	Unstimulated saliva (Early morning)	Luminex MAGPix platform	Inflammatory cytokines IFN-γ IL-10 IL-17 IL-1β IL-4 TNF-α
Raivisto T et al., [46] Finland	Analytical cross-sectional	15–16	*537 participants* Gingivitis (*n* = 166) Subclinical periodontitis (*n* = 345) Periodontally Healthy (*n* = 26)	281 males 256 females	VPI BOP PPD	Stimulated saliva Collection time NR	ELISA IFMA	PGLYRP1 PMN elastase aMMP-8
Rashkova MP et al., [49] Bulgaria	Analytical cross-sectional	15–16	*60 participants* Study group (Gingivitis) (*n* = 30) Control group (Healthy) (*n* = 30)	24 males 36 females	OHI BOP PBI PPD PSR	Unstimulated saliva Morning	ELISA	SIgA
Rinderknecht C et al., [52] Switzerland	Analytical cross-sectional	4–18	*128 participants* Study group (Gingivitis) (*n* = 35) Control group (Healthy) (*n* = 93)	65 males 63 females	Presence or absence of gingivitis	Stimulated saliva Morning (8:00–12:00)	Luminex MAGPix platform	IL-1α, IL-1β, IL-4, IL-5, IL-6, IL-8, IL-10, IL-13, IFN-γ-, IP-10, TNF-α & VEGF-A
Ulker AE et al., [63] Turkey	Analytical cross-sectional	13.6 (Mean age)	*35 participants* Study group (Gingivitis) (*n* = 25) Control group (Healthy) (*n* = 10)	13 males 22 females	PPD CAL PI GI GBI	Unstimulated saliva (10:00–12:00)	LETIA ELISA	Cystatin C IL-1b TNF-α

*aMMP-8 *Active-matrix metalloproteinase-8, *BCA *Bradford Coomassie Assay, *BOP *Bleeding on probing, *CAL *Clinical Attachment Loss, *CPI *Community periodontal index, *CPITN *Community periodontal index of treatment needs, *ELISA * Enzyme-linked immunosorbent assay, *GBI *Gingival Beeding Index, *GI *Gingival Index, *IFMA *Time-resolved immunofluorometric assay, *IFN *Interferon, *Ig *immunoglobulin, *IL *Interleukin, *IP *inducible protein, *LETIA *Latex particle–enhanced turbidimetric immunoassay, *LC-MS/MS *Liquid Chromatography- Mass spectrometry, *MIP *Macrophage inflammatory protein, *MRP *Myeloid-related protein, *MUC-4 *Mucin 4, *NR *Not reported, *OHI *Oral-hygiene index, *PBI *Papilla bleeding index, *PGLYRP1 *Peptidoglycan recognition protein 1, *PI *Plaque Index, *PMN *Polymorphonuclear, *PPD *Periodontal pocket depth, *PSR *Periodontal Screening & Registration, *RBL *Radiographic bone loss, *SIgA *Salivary immunoglobulin A, *sPLA2-IIA *Secretory phospholipase A2 group IIA, *TNF *Tumor Necrosis Factor, *VPI *Visible Plaque Index, *VEGF *vascular endothelial growth factor

Three studies were conducted in the USA [6, 17, 18], followed by two studies each in Brazil [8, 36] and Finland [22, 47]. One study each was conducted in Austria [7], China [14], Bulgaria [49], India [9], Iran [35], Israel [13], Switzerland [52], and Turkey [63] .

Across the 15 studies, a total of 1610 participants were recruited and included 860 males, and 750 females. One study only recruited 32 males as it was conducted in a boys only school [35]. There was a marked variation in the sample size ranging from 30 participants [6, 36] to 252 participants [22].

Most studies conducted the protein analysis on unstimulated saliva (*n* = 11), while only four studies (*n* = 4) collected stimulated saliva [9, 22, 47, 52]. Saliva collection was done in the morning for eight studies (*n* = 8) while the remaining seven studies (*n* = 7) did not report the time of saliva collection [6, 8, 9, 17, 18, 22, 47].

Of the 15 studies, only one study evaluated differentially expressed salivary proteins between periodontally active and periodontally healthy subjects [8], while the remaining 14 studies compared specific salivary proteins between study and control group.

Protein analysis using enzyme linked immunosorbent assay (ELISA) was employed for quantification of salivary proteins in most studies (*n* = 8). Total protein content of saliva was investigated by Bradford method (*n* = 2). Other methods used were Dip stick protein analysis [6], Luminex xMap system [17, 18], Luminex MAGPix platform [36, 52], and Time-resolved immunofluorometric assay (IFMA) [47].

One study carried out a comprehensive analysis of salivary proteome with a focus on evaluating differentially expressed proteins reported in periodontally active and periodontally healthy subjects using Liquid Chromatography coupled with Mass spectrometry (LC-MS/MS) for quantitative proteomic analysis [8].

**Fig. 2:**
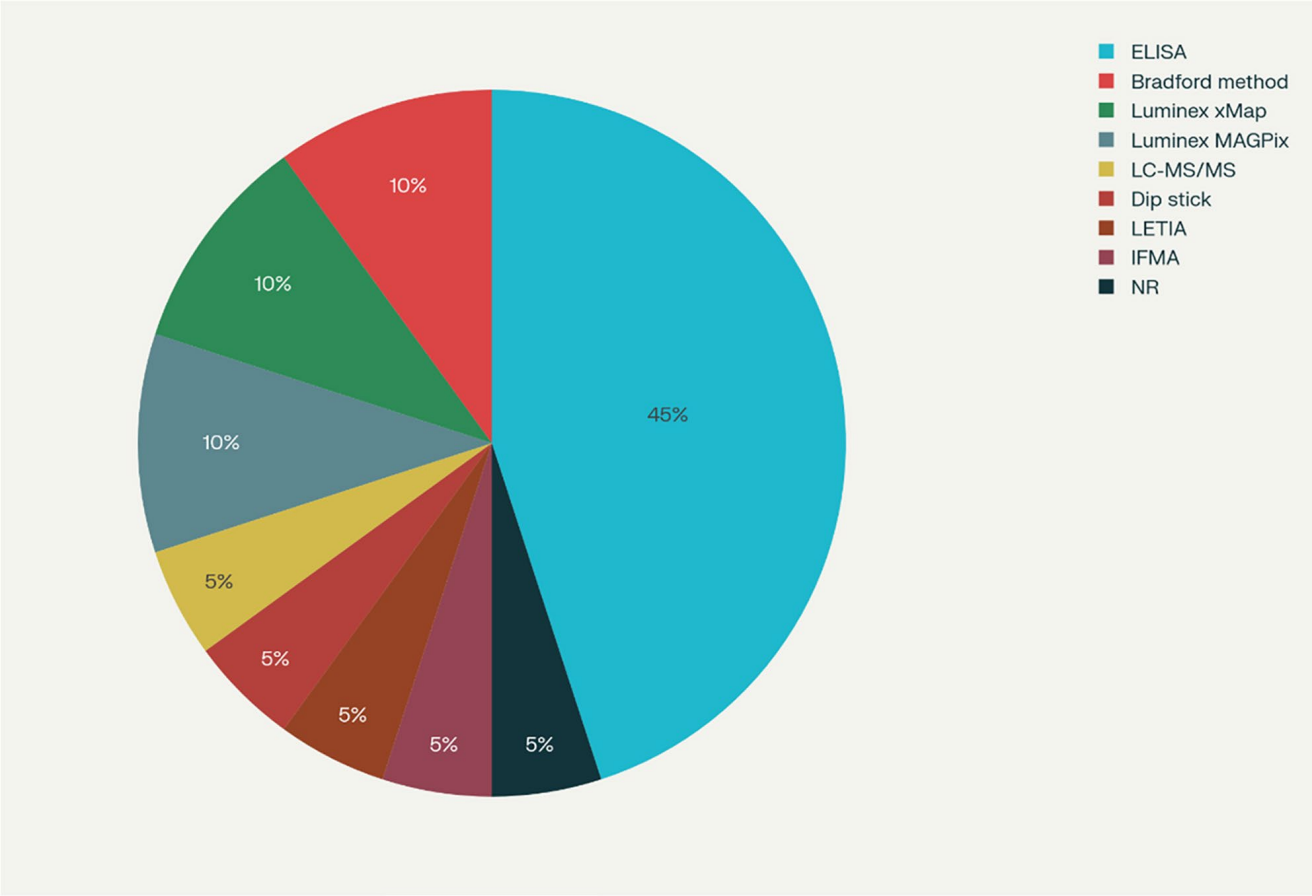
Distribution of salivary proteins quantification methods. Interpretation: ELISA was the most frequently used technique in majority of studies (*n* = 8). Other commonly applied methods included the Bradford method (*n* = 2), Luminex-based systems xMap (*n* = 2) and MAGPix (*n* = 2) followed by Dip stick protein analyses (*n* = 1), IFMA (*n* = 1), and LETIA (*n* = 1). Only one study (*n* = 1) utilized LC-MS/MS for comprehensive proteomic analysis.*ELISA = Enzyme-linked immunosorbent assay; IFMA=Time-resolved immunofluorometric assay; LETIA= Latex particle–enhanced turbidimetric immunoassay; NR = not reported

Different laboratory techniques used to estimate salivary proteins expression in the included studies are summarized as a pie chart depicted in Fig. 2.

### Association between salivary proteins and periodontal disease

The findings of the included studies based on association of salivary proteins with periodontal disease are depicted in Table 3 and summarised below.

**Table 3:** Association of salivary proteins with periodontal status

Study Author	Objective	Salivary protein biomarkers	Key Findings & Conclusions
Bimstein et al., [6]	To determine if dip stick assays of children’s saliva for leukocyte esterase or protein reflect the presence or severity of gingival or periodontal diseases in children	Leukocyte esterase Salivary proteins	• The highest protein value (500 mg/dL) observed in participants with periodontitis. Lower protein values mostly found in healthy participants or those with periodontitis at a single site • Significant differences in the distribution of protein values by the presence/absence of periodontitis (*p* < 0.001) • Leukocyte esterase values not correlated with gingival or periodontal disease
Blufstein, [7]	To measure the salivary level of the inflammatory marker MRP-8/14 in children with gingivitis and periodontally healthy children	MRP-8/14	• Significantly higher levels of MRP-8/14 observed in participants with gingivitis compared to healthy children (*p* = 0.008) • The increased salivary level of MRP-8/14 could be a helpful marker of gingival inflammation
Casarin RCV et al., [8]	To compare the salivary proteomic profile of periodontitis-affected (PA) participants compared to periodontally healthy (PH) participants	Total salivary proteins Differentially expressed salivary proteins	• 19 proteins differentially produced between groups, and five were highly observed in the PA group • Periodontitis patients presented a lower concentration of ANXA1 compared to PH participants • ANXA1 protein suggested as a potential biomarker for periodontitis
Chattopadhyay S et al., [9]	To evaluate the levels of MUC4 and MMP7 in saliva samples taken from periodontitis patients and healthy controls	Total salivary proteins MUC4 MMP7	• MUC4 levels significantly lower in saliva from periodontitis patients compared to healthy controls (*p* < 0.05) • MMP7 levels significantly higher in saliva from periodontitis patients (*p* < 0.05) • MUC4 & MMP7 combination are diagnostic markers for periodontitis
Davidovich E, et al., [13]	To explore the correlation between salivary inflammatory markers with gingival inflammation.	Total salivary proteins Inflammatory cytokines: (IL-6, IL-8, IL-10 &TNFα)	• Elevated levels of total protein identified in individuals with gingival inflammation albeit without statistical significance • IL-6 & TNFα significantly higher among those with high plaque scores (*p* < 0.001) • Salivary cytokines in children may reflect evaluation of oral inflammation
Deng et al., [14]	To explore the association between salivary physicochemical parameters and inflammatory markers	sPLA2-IIA IL-6	• IL-6 output significantly increased in those with more extensive periodontal inflammation (*p* = 0.034) • IL-6 levels negatively correlated with the number of sextants with healthy gingivae and positively correlated with salivary sPLA2-IIA (*p* < 0.05) • Salivary IL-6 levels associated with the extent of periodontal inflammation
Fine DH et al., [18]	To identify cytokine biomarkers that could predict bone loss in LAgP	Total salivary proteins Salivary cytokines: (MIP-1α, MIP-1β, IL-α, IL-1 β & IL-8)	• Cytokine levels associated with acute inflammation (IL-1 β, IL-8, MIP-1α & MIP-1β) elevated in subjects 6–9 months prior to detection of bone loss compared to salivary levels at the time bone loss detected clinically (*p* < 0.01) • MIP-1 α consistently showed elevated levels (13-fold) as a biomarker for bone loss in saliva, 6 months prior to bone loss
Fine DH et al., [17]	To determine if salivary cytokines associated with initial stages of inflammatory periodontitis & bone loss could be detected prior to radiographic evidence of bone loss in a group of LAgP	Salivary cytokines	• The MIP-1 α levels elevated 50-fold in the group that experienced bone loss compared to controls (*p* < 0.001) • IL-1 β statistically significantly elevated in the group with bone loss compared to controls (*p* = 0.01) • MIP-1 α levels could be used as a biomarker of early events in inflammatory-induced periodontal bone loss (LAgP) that precedes radiographic evidence
Heikkinen AM et al., [22]	To investigate how different patient-related risk indicators might be associated with the odds of developing subclinical periodontitis in adolescents.	Total salivary proteins Albumin IgA, IgG, & IgM aMMP-8 PMN elastase	• Subclinical periodontitis in adolescents statistically significantly associated with elevated salivary aMMP-8 but not with PMN elastase (*p* < 0.05) • Elevated total salivary IgG levels associated with lower risk of deterioration of periodontal health in adolescents
Moghadam et al., [35]	To investigate the relationship between the salivary hydroxyproline and the occurrence of gingivitis	Salivary hydroxyproline	• Higher levels of salivary hydroxyproline observed in the gingivitis group compared to the control group with a statistically significant difference (*p* = 0.001) • Salivary hydroxyproline levels can be used as a biomarker in the diagnosis of gingivitis
Monteiro MF et al., [36]	To evaluate the expression of inflammatory cytokines in saliva of children from parents with GAgP compared to children from healthy families	Inflammatory cytokines: (IFN-γ, IL-10, IL-17, IL-1β, IL-4 & TNF-α)	• GAgP group children presented higher PPD & BOP (*p* < 0.05) & lower levels of IL-4 (*p* < 0.05) than the periodontally healthy group • IL-10, IFN-ɣ, IL-17, & IL-4 negatively correlated with the GI, while IL-4 negatively correlated with BOP • Salivary IL-4 conc was a predictor of BOP & gingival inflammation in the children
Raivisto T et al., [47]	To investigate the predictive value of salivary proteins to identify at-risk individuals before the onset of the disease	PGLYRP1 PMN elastase aMMP-8	• Salivary levels of PGLYRP1 & aMMP-8 significantly higher in adolescents with subclinical periodontitis & gingivitis compared to individuals with healthy periodontium (*P* = 0.00394) • PMN elastase levels higher in adolescents with subclinical periodontitis compared to healthy individuals but did not reach significance (*P* > 0.05) • Patients with gingival inflammation (BOP ≥ 20%) showed higher levels of PGLYRP1 in saliva
Rashkova MP et al., [49]	To find the relationship of SIgA to gingival diseases in participants.	SIgA	• The mean SIgA levels higher in participants with healthy gingivae compared to those with gingivitis • A correlation found between SIgA and the plaque biofilm with a level of significance (*p* < 0.05) • SIgA can be considered an imp part of an integrated assessment of oral risk environments
Rinderknecht, [52]	To assess the cytokine levels in healthy participants and assess their association with oral health	IFN-γ, IL-1α, IL-1β, IL-4, IL-5, IL-6, IL-8, IL-10, IL-13, IFN-γ-, IP-10, TNF-α, & VEGF-A	• Increased levels of all cytokines observed in patients with gingivitis with a statistically significant increase for IL-1β, IL-6, IL-8 & IL-10 • 7 out of 10 cytokines (IL-1α, IL-1β, IL-4, IL-5, IP-10, TNF-α, & VEGF-), showed higher median levels in adolescents than in children. • IL-6 & IL-1β levels could predict or confirm oral inflammation
Ulker AE et al., [63]	To evaluate the levels of cystatin C, IL-1 β & TNF- α in the total saliva of periodontally healthy children (PHC) & children with gingivitis (CG)	Cystatin C IL-1 β, TNF- α	• Total saliva Cystatin C & TNF- α levels higher in PHC **(***p* > 0.05) • IL-1 β levels higher in children with gingivitis (CG) **(***p* > 0.05) • Correlations between biochemical markers & clinical parameters seen in the CG group, but not in the PHC group

*ANXA1 *Annexin A1, *aMMP-8 *Active-matrix metalloproteinase-8, *BOP *Bleeding on probing, *GAgP *Generalized Aggressive Periodontitis, *GI *Gingival Index, *IFN *Interferon, *Ig *immunoglobulin, *IL *Interleukin, *IP *inducible protein, *LAgP *localized aggressive periodontitis, *MIP *Macrophage inflammatory protein, *MMP7 *matrix metalloproteinase-7, *MRP *Myeloid-related protein, *MUC-4 *Mucin 4, *PGLYRP1 *Peptidoglycan recognition protein 1, *PI *Plaque Index, *PMN *Polymorphonuclear, *PPD *Periodontal pocket depth, *SIgA *secretory immunoglobulin A, *sPLA2-IIA *Secretory phospholipase A2 group IIA, *TNF *Tumor Necrosis Factor, *VEGF *vascular endothelial growth factor

### Total protein content

Five studies investigated the association of total protein content with periodontal disease. One study reported that the mean total protein level was elevated in individuals with gingival inflammation [13]. However, the findings were not statistically significant. The differences in total salivary protein content between the groups were not reported explicitly by four studies [8, 9, 18, 22].

Significant differences in the distribution of protein values by the presence/absence of periodontitis were reported by one study [6]. The highest protein value (500 mg/dL) was observed in participants with periodontitis.

### Salivary glycoproteins

Three studies reported differential expression of salivary glycoproteins. MUC4 levels were significantly lower in saliva from periodontitis patients compared to healthy controls (*p* < 0.05) [9]. Elevated total salivary IgG levels were associated with lower risk of deterioration of periodontal health in adolescents [22]. The mean SIgA levels were higher in participants with healthy gingivae (*p* < 0.05) compared to those with gingivitis [49].

### Antimicrobial peptides

Only one study investigated total salivary cystatin C; higher levels were observed in periodontally healthy subjects (*p* > 0.05) [63].

### Inflammatory mediators: cytokines

Raised levels of various salivary cytokines were reported to be associated with high plaque scores and periodontal disease by multiple studies. The main results of relevant studies are summarized below:

- IL-6 and TNFα were significantly higher among those with high plaque scores (*P* < 0.001) [13].
- IL-6 output significantly increased in those with more extensive periodontal inflammation (*p* = 0.034). IL-6 levels were negatively correlated with the number of sextants with healthy gingivae and positively correlated with salivary sPLA2-IIA (*p* < 0.05) [14].
- Cytokine levels associated with acute inflammation (IL-1 β, IL-8, MIP-1α & MIP-1β) were elevated in subjects 6–9 months prior to detection of bone loss compared to salivary levels at the time of clinical detection of bone loss (*p* < 0.01). MIP-1 α consistently showed elevated levels (13-fold) as a biomarker for bone loss in saliva, 6 months prior to bone loss [18].
- The levels of MIP-1 α elevated 50-fold in the group that experienced bone loss compared to controls (*p* < 0.001); similarly, IL-1 β were statistically significantly elevated in the group with bone loss compared to controls (*p* = 0.01) [17].
- Negative correlations were identified between the gingival index and IL-10, IFN-ɣ, IL-17, and IL-4. Moreover, IL-4 was negatively correlated with BOP. Participants with generalised aggressive periodontitis presented with lower levels of IL-4 than the periodontally healthy group (*p* < 0.05) [36].
- Increased levels of all cytokines were observed in participants with gingivitis with a statistically significant increase noted for IL-1β, IL-6, IL-8 & IL-10. The concentrations of most of the salivary cytokines were positively correlated with age and the presence of gingivitis and negatively correlated with salivary flow rate. 7 out of 10 cytokines including IL-1α, IL-1β, IL-4, IL-5, IP-10, TNF-α, & VEGF-A, showed higher median levels in adolescents than in children [52].
- TNF- α levels were higher in periodontally healthy participants (*p* > 0.05) while IL-1 β levels were higher in participants with gingivitis (*p* > 0.05) [63].

### Inflammatory mediators: enzymes

- Leukocyte esterase values did not show any correlation with the presence or extent of gingival or periodontal diseases [6].
- Salivary sPLA2-IIA levels were negatively correlated with total salivary bacterial load in adolescents, although this correlation was not significant (*p* > 0.05). Moreover, salivary sPLA2-IIA were positively correlated with salivary IL-6 levels (*p* < 0.05) [14].
- Although increased salivary PMN elastase levels were reported in subclinical periodontitis by two studies, the results were statistically not significant [22, 47].
- Matrix Metallo-proteinases were assessed by three studies. MMP7 levels were significantly higher in saliva from periodontitis patients (*p* < 0.05) [9]. Two studies reported elevated salivary aMMP-8 in participants with subclinical periodontitis (*p* < 0.05) [22, 47].

### Tissue breakdown products

Higher levels of salivary hydroxyproline were observed in the gingivitis group compared to the control group with a statistically significant difference (*p* = 0.001) [35].

### Peptidoglycan recognition protein 1 (PGLYRP1)

Levels of salivary PGLYRP1 were significantly higher in adolescents with subclinical periodontitis and gingivitis compared to individuals with healthy periodontium (*p* = 0.003). PGLYRP1 correlated positively with BOP, PPD, PI, and aMMP8. PGLYRP1 strongly correlated with the plaque levels in the subclinical periodontitis group [47].

### Myeloid-related protein (MRP)-8/14

Significantly higher levels of MRP-8/14 were observed in participants with gingivitis compared to healthy children [7].

### Comprehensive salivary proteomic profile

One study investigated comprehensive salivary proteomic profile [8] 0.19 proteins were differentially expressed between groups with five proteins observed in the periodontally active group. The protein ANXA1 demonstrated the most significant difference in expression levels, showing a 7.1-fold decrease in participants with periodontal disease compared to healthy individuals.

**Fig. 3:**
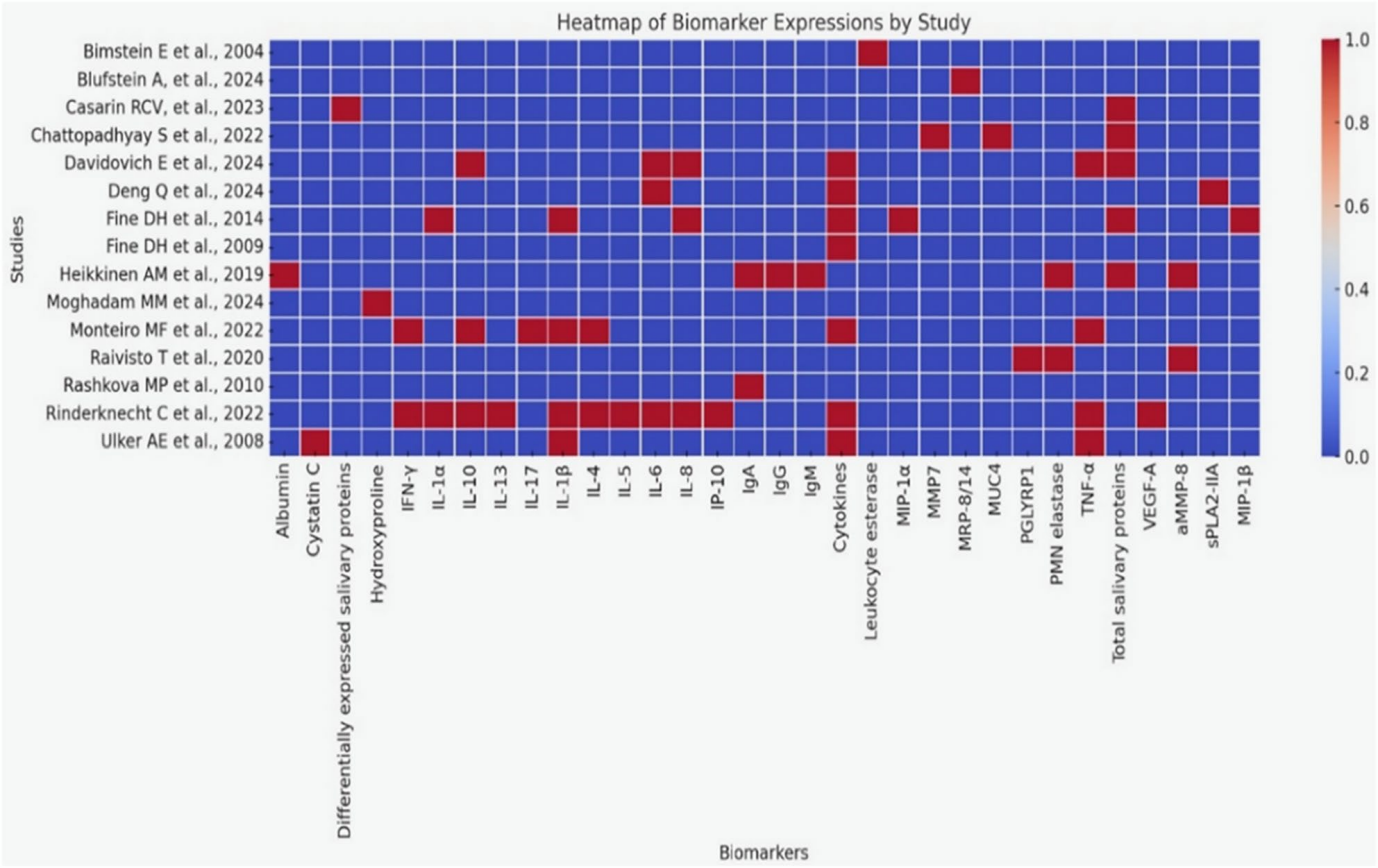
Visual mapping of salivary protein biomarkers across included studies

The expression of salivary protein biomarkers in periodontal disease among children and adolescents is depicted using a heat map in Fig. 3.

## Discussion

To the best of our knowledge, the current study represents a maiden scoping review on the association between salivary proteins and periodontal disease specifically in children and adolescents. Although periodontal disease is less common among young people, it is important to determine the trends and patterns of expression of salivary proteins and inflammatory mediators in this age-group to map the similarities and differences with adults.

The association of salivary protein biomarkers with periodontal disease in children and adolescents is a broad topic and literature on this topic is diverse, emerging, and not fully characterized yet. Although several types of reviews have been published on the association of salivary proteins with periodontal disease, however, they are focused on the adult population, and most studies do not include children or adolescents. Therefore, a scoping review was considered to be appropriate to comprehensively map evidence about salivary protein biomarkers and periodontal disease risk in children and adolescents. Given the diverse studies published on this topic, conducting a systematic review was not feasible as it was difficult to quantitatively compare levels of different proteins between the groups due to heterogeneity in diagnostic criteria and inconsistencies in reporting expression of different salivary proteins. Evidence from the literature suggests that a scoping review is suitable to identify available evidence, and research gaps without requiring rigorous quality appraisal [37]. This scoping review captured the full scope of evidence and may serve to inform future focused research including systematic reviews.

The findings of this scoping review support their potential utility as non-invasive diagnostic markers for detecting early inflammatory changes and predicting disease progression, particularly in conditions like localized aggressive periodontitis in children and adolescents. Overall, the results of this scoping review indicate that up to 66% of salivary proteins are significantly associated with periodontal disease, highlighting their potential as a biomarker of periodontal disease. However, the findings also underscore the complex nature of periodontal disease in children and adolescents and the multifactorial interplay between different protein markers.

The most remarkable results related to inflammatory cytokines (IL-6, IL-1β, TNF-α, MIP-1α, and MMP7) which consistently demonstrated a strong association with both gingival inflammation and periodontal tissue destruction. A recent study on adult patients also reported raised levels of IL-6, IL-1β in periodontitis [51]. However, the total protein content in saliva, although elevated in some cases of periodontal disease, did not consistently correlate with disease status, suggesting that it may not serve as a reliable standalone biomarker. These findings corroborate with a previous study on young adults which did not find any significant correlation between total protein content and aggressive periodontitis [1]. Cystatin C has a protective role to prevent periodontal breakdown, and its expression decreases after treatment, as bone homeostasis is achieved. It has been previously reported that cystatin C levels in serum and crevicular fluid increase with periodontal disease progression [56]. However, the results of the current review suggest that while cystatin C showed elevated levels in healthy individuals, the differences were not statistically significant.

Salivary glycoproteins, including MUC4 and SIgA, displayed potential in distinguishing between healthy and diseased states, indicating their role in maintaining oral health and potentially mitigating inflammation, as reported in previous studies [54]. Additionally, proteins related to tissue breakdown (hydroxyproline) and specific immune components (PGLYRP1) were significantly elevated in inflammatory conditions, pointing towards their potential as indicators of tissue degradation and immune response, respectively. The integration of comprehensive salivary proteomic profiling further revealed distinct differences in protein expression between periodontally healthy and affected individuals, with ANXA1 protein emerging as a promising marker for periodontal disease [8].

### Limitations

This scoping review identified several gaps in the literature on salivary protein expression in children and adolescents with periodontal disease. Firstly, the studies used heterogenous research designs, and targeted different proteins. Saliva collection methods, timings, and laboratory techniques for measuring salivary proteins and inflammatory mediators also varied. It is also important to reiterate that the levels of salivary proteins can vary with the type of saliva collection i.e., whole saliva (stimulated vs. unstimulated), and possibly the time of collection (Al [21]). Moreover, some of the included studies were undertaken prior to the introduction of the updated guidelines on the classification of periodontal disease published in 2017 by the European Federation of Periodontology (EFP) and American Academy of Periodontology (AAP) [19, 43]. Therefore, the diagnosis of periodontal disease in studies carried out before the introduction of these guidelines may not align with the current classification and pose potential limitations to the interpretation of the results. Differences in study design, sample size, demographic characteristics, disease classification criteria, and methods for protein analysis may have contributed to the variability in findings. Second, the cross-sectional nature of most studies means that temporal relationships between salivary protein biomarker levels and disease progression could not be assessed. Longitudinal studies are needed to clarify whether changes in biomarker levels precede or follow disease onset and progression.

Additionally, the lack of standardization in salivary protein biomarker measurement methods and clinical assessment limits the comparability of results. Variations in saliva collection protocols, analytical techniques, and data interpretation could introduce bias and affect the reliability of biomarker comparisons across studies. Only one study investigated the salivary proteomic profile comprehensively while the remaining 14 studies only focussed on specific biomarkers. None of the studies provided details of calibration of reagents and equipment used for protein analysis explicitly which may impact on reproducibility of these techniques. Lastly, publication bias may be present, as studies with significant findings are more likely to be published compared to those reporting null results. This may have skewed the overall conclusions regarding the association between salivary protein biomarkers and periodontal disease.

These gaps in the included studies underscore the need to standardize the diagnostic criteria as per the current periodontology guidelines, and methods used for saliva collection, and analysis of salivary proteins to determine their correlation with periodontal disease in a reliable manner.

### Recommendations

Although some progress has been made to explore the association of salivary proteins with periodontal disease in children and adolescents, it is important to advance the research by evaluating the sensitivity and specificity of salivary protein biomarkers by utilizing persistent development of technologies including artificial intelligence (AI) [2]. The use of AI has the potential to significantly enhance the investigation of correlations between salivary protein biomarkers and periodontal disease by enabling the analysis of complex, high-dimensional data and uncovering patterns that might be difficult to detect using traditional statistical methods.

Further research is needed to fully understand the role of salivary proteins, especially antimicrobial proteins in the pathogenesis of oral diseases and to validate their use as potential biomarkers. Future research should employ large-scale longitudinal study designs to explore the temporal correlations between salivary proteins and periodontal disease. This may help to determine if expression of salivary proteins indicates a protective response or a risk factor for periodontal disease in children and adolescents. Moreover, standardized saliva collection procedures and consistent methodological protocols should be adopted to enable meaningful comparisons between future studies. The afore-mentioned improvements in research protocols may unravel the complexities of expression of salivary proteins and specifically how they can be used for risk prediction and monitoring of periodontal disease.

## Conclusions

The synthesis of studies included in this review shows a complex, albeit preliminary, association between salivary protein profiles and periodontal status in young populations. The interpretations are tempered by significant heterogeneity and methodological limitations across the included studies. Observed trends, such as the elevation of specific markers (MIP-1α, IL-6, MMP7) in disease and glycoproteins in health, must be interpreted with caution due to variations in study design, sample collection, and analytical techniques. The inconsistent findings for total protein and cystatin C further underscore the preliminary nature of these results. Therefore, while salivary proteins represent a promising avenue for future research, their role as reliable biomarkers for diagnosis and monitoring cannot be confirmed from the existing body of evidence, which highlights a need for more standardized investigations with longitudinal data.

## Supplementary Information

Supplementary Material 1.

## Data Availability

The datasets analyzed during the current study are available from the corresponding author on reasonable request.
