# Supplementary material for: Association of salivary proteins with periodontal disease in children and adolescents- a scoping review
Source: BMC Oral Health. 2026 Feb 28;26:592. doi: 10.1186/s12903-026-08005-2 (PMC13059474; doi:10.1186/s12903-026-08005-2)
Supplement: Supplementary file 1 — Supplementary Material 1. [file 12903_2026_8005_MOESM1_ESM.docx]

**Table Is: Preferred Reporting Items for Systematic reviews and Meta-Analyses extension for Scoping Reviews (PRISMA-ScR) Checklist**

| **SECTION** | **ITEM** | **PRISMA-ScR CHECKLIST ITEM** | **REPORTED ON PAGE #** |
| --- | --- | --- | --- |
| **TITLE** | | | |
| Title | 1 | Identify the report as a scoping review. | 1 |
| **ABSTRACT** | | | |
| Structured summary | 2 | Provide a structured summary that includes (as applicable): background, objectives, eligibility criteria, sources of evidence, charting methods, results, and conclusions that relate to the review questions and objectives. | 2 |
| **INTRODUCTION** | | | |
| Rationale | 3 | Describe the rationale for the review in the context of what is already known. Explain why the review questions/objectives lend themselves to a scoping review approach. | 5 |
| Objectives | 4 | Provide an explicit statement of the questions and objectives being addressed with reference to their key elements (e.g., population or participants, concepts, and context) or other relevant key elements used to conceptualize the review questions and/or objectives. | 5-6 |
| **METHODS** | | | |
| Protocol and registration | 5 | Indicate whether a review protocol exists; state if and where it can be accessed (e.g., a Web address); and if available, provide registration information, including the registration number. | 6 |
| Eligibility criteria | 6 | Specify characteristics of the sources of evidence used as eligibility criteria (e.g., years considered, language, and publication status), and provide a rationale. | 7 |
| Information sources* | 7 | Describe all information sources in the search (e.g., databases with dates of coverage and contact with authors to identify additional sources), as well as the date the most recent search was executed. | 7-8 |
| Search | 8 | Present the full electronic search strategy for at least 1 database, including any limits used, such that it could be repeated. | 7-8 |
| Selection of sources of evidence† | 9 | State the process for selecting sources of evidence (i.e., screening and eligibility) included in the scoping review. | 9 |
| Data charting process‡ | 10 | Describe the methods of charting data from the included sources of evidence (e.g., calibrated forms or forms that have been tested by the team before their use, and whether data charting was done independently or in duplicate) and any processes for obtaining and confirming data from investigators. | 9 |
| Data items | 11 | List and define all variables for which data were sought and any assumptions and simplifications made. | 9 |
| Critical appraisal of individual sources of evidence§ | 12 | If done, provide a rationale for conducting a critical appraisal of included sources of evidence; describe the methods used and how this information was used in any data synthesis (if appropriate). | NA |
| Synthesis of results | 13 | Describe the methods of handling and summarizing the data that were charted. | 9-10 |
| **RESULTS** | | | |
| Selection of sources of evidence | 14 | Give numbers of sources of evidence screened, assessed for eligibility, and included in the review, with reasons for exclusions at each stage, ideally using a flow diagram. | 11-12 |
| Characteristics of sources of evidence | 15 | For each source of evidence, present characteristics for which data were charted and provide the citations. | 12-18 |
| Critical appraisal within sources of evidence | 16 | If done, present data on critical appraisal of included sources of evidence (see item 12). | NA |
| Results of individual sources of evidence | 17 | For each included source of evidence, present the relevant data that were charted that relate to the review questions and objectives. | 12-26 |
| Synthesis of results | 18 | Summarize and/or present the charting results as they relate to the review questions and objectives. | 12-26 |
| **DISCUSSION** | | | |
| Summary of evidence | 19 | Summarize the main results (including an overview of concepts, themes, and types of evidence available), link to the review questions and objectives, and consider the relevance to key groups. | 27-29 |
| Limitations | 20 | Discuss the limitations of the scoping review process. | 29-30 |
| Conclusions | 21 | Provide a general interpretation of the results with respect to the review questions and objectives, as well as potential implications and/or next steps. | 31 |
| **FUNDING** | | | |
| Funding | 22 | Describe sources of funding for the included sources of evidence, as well as sources of funding for the scoping review. Describe the role of the funders of the scoping review. | No funding received for the study |

JBI = Joanna Briggs Institute; PRISMA-ScR = Preferred Reporting Items for Systematic reviews and Meta-Analyses extension for Scoping Reviews.

* Where *sources of evidence* (see second footnote) are compiled from, such as bibliographic databases, social media platforms, and Web sites.

† A more inclusive/heterogeneous term used to account for the different types of evidence or data sources (e.g., quantitative and/or qualitative research, expert opinion, and policy documents) that may be eligible in a scoping review as opposed to only studies. This is not to be confused with *information sources* (see first footnote).

‡ The frameworks by Arksey and O’Malley (6) and Levac and colleagues (7) and the JBI guidance (4, 5) refer to the process of data extraction in a scoping review as data charting*.*

§ The process of systematically examining research evidence to assess its validity, results, and relevance before using it to inform a decision. This term is used for items 12 and 16 instead of "risk of bias" (which is more applicable to systematic reviews of interventions) to include and acknowledge the various sources of evidence that may be used in a scoping review (e.g., quantitative and/or qualitative research, expert opinion, and policy document).

*From:* Tricco AC, Lillie E, Zarin W, O'Brien KK, Colquhoun H, Levac D, et al. PRISMA Extension for Scoping Reviews (PRISMAScR): Checklist and Explanation. Ann Intern Med. 2018;169:467–473. [doi: 10.7326/M18-0850](http://annals.org/aim/fullarticle/2700389/prisma-extension-scoping-reviews-prisma-scr-checklist-explanation).

**Table IIs: Search terms and Data bases used**

| **Database** |  | **Query** | **Results** |
| --- | --- | --- | --- |
| PubMed | #1 | ((((((((((((((((((((((((("saliva proteins") OR ("saliva peptides")) OR ("saliva proteome")) OR ("Salivary Proteins and Peptides"[Mesh])) OR ("salivary protein biomarkers")) OR ("salivary proteomic profile")) OR ("salivary protein expression")) OR ("total salivary proteins")) OR ("Mucin-5B"[Mesh])) OR ("Salivary Proline-Rich Proteins"[Mesh])) OR ("Salivary alpha-Amylases"[Mesh])) OR ("Histatins"[Mesh])) OR ("Salivary Cystatins"[Mesh])) OR ("Matrix Metalloproteinase 8"[Mesh])) OR ("Fibronectins"[Mesh])) OR ("Antimicrobial Cationic Peptides"[Mesh])) OR ("salivary mucins")) OR ("salivary IgA")) OR ("salivary statherin")) OR (salivary defensins)) OR (salivary cathelicidins)) OR (salivary human lysozyme)) OR ("salivary lactoferrin")) OR ("salivary glycoproteins")) OR (salivary cytokines)) OR (salivary peroxidase) | 77,144 |
|  | #2 | (((((((("Periodontitis"[Mesh]) OR ("Gingivitis"[Mesh])) OR ("Periodontal Pocket"[Mesh])) OR ("periodontal disease")) OR ("gingival disease")) OR ("periodontal inflammation")) OR ("gingival inflammation")) OR ("gum disease")) | 64,091 |
|  | #3 | #1 **AND** #2 | 1,334 |
|  |  | (((((((((((((((((((((((((("saliva proteins") OR ("saliva peptides")) OR ("saliva proteome")) OR ("Salivary Proteins and Peptides"[Mesh])) OR ("salivary protein biomarkers")) OR ("salivary proteomic profile")) OR ("salivary protein expression")) OR ("total salivary proteins")) OR ("Mucin-5B"[Mesh])) OR ("Salivary Proline-Rich Proteins"[Mesh])) OR ("Salivary alpha-Amylases"[Mesh])) OR ("Histatins"[Mesh])) OR ("Salivary Cystatins"[Mesh])) OR ("Matrix Metalloproteinase 8"[Mesh])) OR ("Fibronectins"[Mesh])) OR ("Antimicrobial Cationic Peptides"[Mesh])) OR ("salivary mucins")) OR ("salivary IgA")) OR ("salivary statherin")) OR (salivary defensins)) OR (salivary cathelicidins)) OR (salivary human lysozyme)) OR ("salivary lactoferrin")) OR ("salivary glycoproteins")) OR (salivary cytokines)) OR (salivary peroxidase)) **AND** ((((((((("Periodontitis"[Mesh]) OR ("Gingivitis"[Mesh])) OR ("Periodontal Pocket"[Mesh])) OR ("periodontal disease")) OR ("gingival disease")) OR ("periodontal inflammation")) OR ("gingival inflammation")) OR ("gum disease"))) |  |
|  | #4 | Filters | 1,127 |
|  |  | (((((((((((((((((((((((((("saliva proteins") OR ("saliva peptides")) OR ("saliva proteome")) OR ("Salivary Proteins and Peptides"[Mesh])) OR ("salivary protein biomarkers")) OR ("salivary proteomic profile")) OR ("salivary protein expression")) OR ("total salivary proteins")) OR ("Mucin-5B"[Mesh])) OR ("Salivary Proline-Rich Proteins"[Mesh])) OR ("Salivary alpha-Amylases"[Mesh])) OR ("Histatins"[Mesh])) OR ("Salivary Cystatins"[Mesh])) OR ("Matrix Metalloproteinase 8"[Mesh])) OR ("Fibronectins"[Mesh])) OR ("Antimicrobial Cationic Peptides"[Mesh])) OR ("salivary mucins")) OR ("salivary IgA")) OR ("salivary statherin")) OR (salivary defensins)) OR (salivary cathelicidins)) OR (salivary human lysozyme)) OR ("salivary lactoferrin")) OR ("salivary glycoproteins")) OR (salivary cytokines)) OR (salivary peroxidase)) AND ((((((((("Periodontitis"[Mesh]) OR ("Gingivitis"[Mesh])) OR ("Periodontal Pocket"[Mesh])) OR ("periodontal disease")) OR ("gingival disease")) OR ("periodontal inflammation")) OR ("gingival inflammation")) OR ("gum disease"))) AND ((humans[Filter]) AND (english[Filter])) |  |
| SCOPUS | #1 | TITLE-ABS-KEY("saliva proteins" OR "saliva peptides" OR "saliva proteome" OR "Salivary proteins and peptides" OR "salivary protein biomarkers" OR "salivary proteomic profile" OR "salivary protein expression" OR "total salivary proteins" OR "Mucin-5B" OR "Salivary Proline-Rich Proteins" OR "Salivary alpha-Amylases" OR "Histatins" OR "Salivary Cystatins" OR "Matrix Metalloproteinase 8" OR "Fibronectins" OR "Antimicrobial Cationic Peptides" OR "salivary mucins" OR "salivary IgA" OR "salivary statherin" OR "salivary defensins" OR "salivary cathelicidins" OR "salivary human lysozyme" OR "salivary lactoferrin" OR "salivary glycoproteins" OR "salivary cytokines” OR "salivary peroxidase") | 91,477 |
|  | #2 | TITLE-ABS-KEY("Periodontitis" OR "Gingivitis" OR "Periodontal Pocket" OR "periodontal disease" OR "gingival disease" OR "periodontal inflammation" OR "gingival inflammation" OR "gum disease") | 129,920 |
|  | #3 | #1 **AND** #2 | 1,631 |
|  |  | ( TITLE-ABS-KEY ( "saliva proteins" OR "saliva peptides" OR "saliva proteome" OR "Salivary proteins and peptides" OR "salivary protein biomarkers" OR "salivary proteomic profile" OR "salivary protein expression" OR "total salivary proteins" OR "Mucin-5B" OR "Salivary Proline-Rich Proteins" OR "Salivary alpha-Amylases" OR "Histatins" OR "Salivary Cystatins" OR "Matrix Metalloproteinase 8" OR "Fibronectins" OR "Antimicrobial Cationic Peptides" OR "salivary mucins" OR "salivary IgA" OR "salivary statherin" OR "salivary defensins" OR "salivary cathelicidins" OR "salivary human lysozyme" OR "salivary lactoferrin" OR "salivary glycoproteins" OR "salivary cytokines" OR "salivary peroxidase" ) ) **AND** ( TITLE-ABS-KEY ( "Periodontitis" OR "Gingivitis" OR "Periodontal Pocket" OR "periodontal disease" OR "gingival disease" OR "periodontal inflammation" OR "gingival inflammation" OR "gum disease" ) ) |  |
|  | #4 | Filters | 1,248 |
|  |  | ( TITLE-ABS-KEY ( "saliva proteins" OR "saliva peptides" OR "saliva proteome" OR "Salivary proteins and peptides" OR "salivary protein biomarkers" OR "salivary proteomic profile" OR "salivary protein expression" OR "total salivary proteins" OR "Mucin-5B" OR "Salivary Proline-Rich Proteins" OR "Salivary alpha-Amylases" OR "Histatins" OR "Salivary Cystatins" OR "Matrix Metalloproteinase 8" OR "Fibronectins" OR "Antimicrobial Cationic Peptides" OR "salivary mucins" OR "salivary IgA" OR "salivary statherin" OR "salivary defensins" OR "salivary cathelicidins" OR "salivary human lysozyme" OR "salivary lactoferrin" OR "salivary glycoproteins" OR "salivary cytokines" OR "salivary peroxidase" ) ) AND ( TITLE-ABS-KEY ( "Periodontitis" OR "Gingivitis" OR "Periodontal Pocket" OR "periodontal disease" OR "gingival disease" OR "periodontal inflammation" OR "gingival inflammation" OR "gum disease" ) ) AND ( LIMIT-TO ( EXACTKEYWORD , "Human" ) ) AND ( LIMIT-TO ( LANGUAGE , "English" ) ) |  |

| EMBASE | #1 | 'saliva protein'/exp OR 'saliva peptides' OR 'saliva proteome' OR 'salivary proteins'/exp OR 'salivary peptides' OR 'salivary protein biomarkers' OR 'salivary proteomic profile' OR 'salivary protein expression' OR 'total salivary proteins' OR 'mucin 5b'/exp OR 'proline rich protein'/exp OR 'salivary alpha-amylases'/exp OR 'histatins'/exp OR 'cystatin s'/exp OR 'matrix metalloproteinase 8'/exp OR 'fibronectins'/exp OR 'antimicrobial cationic peptides'/exp OR 'salivary mucins' OR 'salivary iga' OR 'salivary statherin' OR 'salivary defensins' OR 'salivary cathelicidins' OR 'salivary human lysozyme' OR 'salivary lactoferrin' OR 'salivary glycoproteins' OR 'salivary cytokines' OR 'salivary peroxidase' | 80,148 |
| --- | --- | --- | --- |
|  | #2 | 'periodontitis'/exp OR 'gingivitis'/exp OR 'periodontal pocket'/exp OR 'periodontal disease'/exp OR 'gingival disease' OR 'periodontal inflammation'/exp OR 'periodontal inflammation' OR 'gingival inflammation'/exp OR 'gingival inflammation' OR 'gum disease'/exp OR 'gum disease' | 137,081 |
|  | #3 | #1 **AND** #2 | 1,593 |
|  |  | ('saliva protein'/exp OR 'saliva peptides' OR 'saliva proteome' OR 'salivary proteins'/exp OR 'salivary peptides' OR 'salivary protein biomarkers' OR 'salivary proteomic profile' OR 'salivary protein expression' OR 'total salivary proteins' OR 'mucin 5b'/exp OR 'proline rich protein'/exp OR 'salivary alpha-amylases'/exp OR 'histatins'/exp OR 'cystatin s'/exp OR 'matrix metalloproteinase 8'/exp OR 'fibronectins'/exp OR 'antimicrobial cationic peptides'/exp OR 'salivary mucins' OR 'salivary iga' OR 'salivary statherin' OR 'salivary defensins' OR 'salivary cathelicidins' OR 'salivary human lysozyme' OR 'salivary lactoferrin' OR 'salivary glycoproteins' OR 'salivary cytokines' OR ‘salivary peroxidase') **AND** ('periodontitis'/exp OR 'gingivitis'/exp OR 'periodontal pocket'/exp OR 'periodontal disease'/exp OR 'gingival disease' OR 'periodontal inflammation'/exp OR 'periodontal inflammation' OR 'gingival inflammation'/exp OR 'gingival inflammation' OR 'gum disease'/exp OR 'gum disease') |  |
|  | #4 | Filters | 1,333 |
|  |  | ('saliva protein'/exp OR 'saliva peptides' OR 'saliva proteome' OR 'salivary proteins'/exp OR 'salivary peptides' OR 'salivary protein biomarkers' OR 'salivary proteomic profile' OR 'salivary protein expression' OR 'total salivary proteins' OR 'mucin 5b'/exp OR 'proline rich protein'/exp OR 'salivary alpha-amylases'/exp OR 'histatins'/exp OR 'cystatin s'/exp OR 'matrix metalloproteinase 8'/exp OR 'fibronectins'/exp OR 'antimicrobial cationic peptides'/exp OR 'salivary mucins' OR 'salivary iga' OR 'salivary statherin' OR 'salivary defensins' OR 'salivary cathelicidins' OR 'salivary human lysozyme' OR 'salivary lactoferrin' OR 'salivary glycoproteins' OR 'salivary cytokines' OR 'salivary peroxidase') AND ('periodontitis'/exp OR 'gingivitis'/exp OR 'periodontal pocket'/exp OR 'periodontal disease'/exp OR 'gingival disease' OR 'periodontal inflammation'/exp OR 'periodontal inflammation' OR 'gingival inflammation'/exp OR 'gingival inflammation' OR 'gum disease'/exp OR 'gum disease') AND [humans]/lim AND [english]/lim |  |

| Web of Science | #1 | **ALL=( ("saliva proteins" OR "saliva peptides" OR "saliva proteome" OR "Salivary Proteins and Peptides" OR "salivary protein biomarkers" OR "salivary proteomic profile" OR "salivary protein expression" OR "total salivary proteins" OR "Mucin-5B" OR "Salivary Proline-Rich Proteins" OR "Salivary alpha-Amylases" OR "histatin" OR "Salivary Cystatins" OR "Matrix Metalloproteinase 8" OR "Fibronectins" OR "Antimicrobial Cationic Peptides" OR "salivary mucins" OR "salivary IgA" OR "salivary statherin" OR "salivary defensins" OR "salivary cathelicidins" OR "salivary human lysozyme" OR "salivary lactoferrin" OR "salivary glycoproteins" OR "salivary cytokines" OR "salivary peroxidase"))** | 5,356 |
| --- | --- | --- | --- |
|  | #2 | ALL=(("Periodontitis" OR "Gingivitis" OR "Periodontal Pocket" OR "periodontal disease" OR "gingival disease" OR "periodontal inflammation" OR "gingival inflammation" OR "gum disease")) | 64,343 |
|  | #3 | #1 **AND** #2 | 666 |
|  | #3 | Filters | 662 |
| Google Scholar | #1 | “saliva proteins” **AND** “periodontitis”  <https://scholar.google.com/scholar?hl=en&as_sdt=0%2C5&q=%E2%80%9Csaliva+proteins%E2%80%9D+AND+%E2%80%9Cperiodontitis%E2%80%9D&btnG=> | 690 |

**Table IIIs: List of excluded articles and the reasons for exclusion**

| **NO** | **AUTHOR/YEAR** | **TITLE OF THE STUDY** | **REASON FOR EXCLUSION** |
| --- | --- | --- | --- |
| 1 | Abdullameer MA et al., 2023 | Salivary interleukin-1β as a biomarker to differentiate between periodontal health, gingivitis, and periodontitis | Could not retrieve full text |
| 2 | Acquier AB et al., 2015 | Comparison of salivary levels of mucin and amylase and their relation with clinical parameters obtained from patients with aggressive and chronic periodontal disease | Different study population |
| 3 | Afacan B, et al.,1980 | Alarm anti-protease trappin-2 negatively correlates with proinflammatory cytokines in patients with periodontitis | Different study population |
| 4 | Afacan B et al.,2019 | Gingival crevicular fluid and salivary HIF-1α, VEGF, and TNF-α levels in periodontal health and disease. | Different study population |
| 5 | Aji NRAS et al.,2024 | aMMP-8 POCT vs. Other Potential Biomarkers in Chair-Side Diagnostics and Treatment Monitoring of Severe Periodontitis | Different study population |
| 6 | Akhi R et al., 2022 | Salivary IgA antibody to malondialdehyde-acetaldehyde associates with mild periodontal pocket depth. | Wrong Study Population |
| 7 | Alhammadi A et al.,2023 | Salivary macrophage chemokines as potential biomarkers of gingivitis. | Different study population |
| 8 | Armitage GC., 2000 | Analysis of gingival crevice fluid and risk of progression of periodontitis. | Review |
| 9 | Baeza M et al .,2016 | Diagnostic accuracy for apical and chronic periodontitis biomarkers in gingival crevicular fluid: an exploratory study. | Different study population |
| 10 | Baliban RC et al.,2011 | Novel protein identification methods for biomarker discovery via a proteomic analysis of periodontally healthy and diseased gingival crevicular fluid samples | Different study population |
| 11 | Baron AC et al.,1999 | Cysteine protease inhibitory activity and levels of salivary cystatins in whole saliva of periodontally diseased patients. | Different study population |
| 12 | Belstrøm D et al., 2017 | Salivary cytokine levels in early gingival inflammation | Different study population |
| 13 | Bildt MM et al., 2008 | Collagenolytic fragments and active gelatinase complexes in periodontitis. | Different study population |
| 14 | Bostanci N et al., 2017 | Contribution of proteomics to our understanding of periodontal inflammation | Review |
| 15 | Buduneli N et al., 2011 | Host-derived diagnostic markers related to soft tissue destruction and bone degradation in periodontitis | Review |
| 16 | Caffesse RG et al., 1993 | Polypeptide growth factors and attachment proteins in periodontal wound healing and regeneration | Review |
| 17 | Castagnola M et al., 2001 | Determination of the human salivary peptides histatins 1, 3, 5 and statherin by high-performance liquid chromatography and by diode-array detection | Different aim |
| 18 | Chung WO et al., 2007 | Expression of defensins in gingiva and their role in periodontal health and disease | Review |
| 19 | Costa LCM et al., 2018 | Gingival crevicular fluid levels of human beta-defensin 1 in individuals with and without chronic periodontitis | Different study population |
| 20 | da Silva CVF et al., 2023 | Comparative proteomics of saliva of healthy and gingivitis individuals from Rio de Janeiro | Different study population |
| 21 | Dale BA et al., 2001 | Localized antimicrobial peptide expression in human gingiva | Different aim |
| 22 | De Smet K et al., 2005 | Human antimicrobial peptides: defensins, cathelicidins and histatins | Review |
| 23 | de Souza-Gugelmin MC et al., 1995 | Creation of the gingival immunologic defense index (GIDI) to evaluate the immunological potential of the gingiva and the possible risk for periodontal disease | Full text not available |
| 24 | De-Gennaro LA et al., 2006 | Autoantibodies directed to extracellular matrix components in patients with different clinical forms of periodontitis | Different study population |
| 25 | Embery G et al., 1994 | Gingival crevicular fluid: biomarkers of periodontal tissue activity | Review |
| 26 | Ersin Kalkan R et al., 2018 | Salivary fetuin-A, S100A12, and high-sensitivity C-reactive protein levels in periodontal diseases | Different study population |
| 27 | Fábián TK et al., 2012 | Salivary defense proteins: their network and role in innate and acquired oral immunity | Review |
| 28 | Ford PJ et al., 2010 | Immunological differences and similarities between chronic periodontitis and aggressive periodontitis | Review |
| 29 | Front E et al., 2013 | Salivary biomarker analysis complementing regular clinical examination | Different study population |
| 30 | Galunska et al., 2024 | Gingival status and prophylactic oral hygiene measures modulate salivary amino acids’ profile in children with plaque-induced gingivitis | Different aim |
| 31 | Gonçalves Lda R et al., 2010 | Comparative proteomic analysis of whole saliva from chronic periodontitis patients | Different study population |
| 32 | Gonçalves PF et al., 2013 | Periodontal treatment reduces matrix metalloproteinase levels in localized aggressive periodontitis | Different aim |
| 33 | Görgülü NG et al., 2022 | Effect of non-surgical periodontal treatment on salivary and serum biomarkers in Stage III Grade B and C periodontitis | Different study population |
| 34 | N. G. GÖRGÜLÜ et al., 2022 | Salivary MMP-8, MAF, and IL-34 levels in non-surgical periodontal treatment of gingivitis patients. | Conference paper |
| 35 | Groenink J et al., 1999 | Salivary lactoferrin and low-Mr mucin MG2 in Actinobacillus actinomycetemcomitans-associated periodontitis | Different study population |
| 36 | Güncü GN et al., 2015 | Salivary Antimicrobial Peptides in Early Detection of Periodontitis. | Review |
| 37 | Gupta S et al., 2021 | Linking oral microbial proteolysis to aMMP-8 PoC diagnostics along with the stage and grade of periodontitis: A cross-sectional study | Short communication |
| 38 | Gursoy UK et al., 2016 | Associations Between Salivary Bone Metabolism Markers and Periodontal Breakdown | Different study population |
| 39 | Güven O et al., 1982 | Salivary IgA in periodontal disease | Full text not available |
| 40 | Hadzic Z et al ., 2021 | Salivary Interleukin-6 Levels in Patients with Periodontitis Stage IV | Different study population |
| 41 | Hägewald SJ et al., 2003 | Salivary IgA in response to periodontal treatment | Different study population |
| 42 | Hartenbach FARR et al., 2020 | Proteomic analysis of whole saliva in chronic periodontitis | Different study population |
| 43 | Hayakawa H et al., 1994 | Collagenase activity and tissue inhibitor of metalloproteinases-1 (TIMP-1) content in human whole saliva from clinically healthy and periodontally diseased subjects | Different study population |
| 44 | Heikkinen AM et al., 2024 | Prevention of Initial Periodontitis Is an Investment in the Future | Different aim |
| 45 | Heikkinen AM et al., 2016 | Pilot Study on Oral Health Status as Assessed by an Active-Matrix Metalloproteinase-8 Chairside Mouthrinse Test in Adolescents | Different aim |
| 46 | Heikkinen AM et al., 2022 | Implementing of aMMP-8 point-of-care test with a modified new disease classification in Finnish adolescent cohorts | Different aim |
| 47 | Henskens YM et al., 1993 | Cystatin C levels of whole saliva are increased in periodontal patients. | Study population not mentioned |
| 48 | Henskens YM et al., 1993 | Protein, albumin and cystatin concentrations in saliva of healthy subjects and of patients with gingivitis or periodontitis. | Full text not available |
| 49 | Henskens, Y. M. C et al., 1995 | Salivary cystatins and their relation to periodontal disease | Abstract not available |
| 50 | Hernández M et al., 2021 | Active MMP-8 Quantitative Test as an Adjunctive Tool for Early Diagnosis of Periodontitis | Different study population |
| 51 | Hong I et al., 2020 | Oral Fluid Biomarkers for Diagnosing Gingivitis in Human: A Cross-Sectional Study | Different study population |
| 52 | Hormia M et al., 1993 | Increased rate of salivary epidermal growth factor secretion in patients with juvenile periodontitis. | Different study population |
| 53 | Huynh AH et al., 2015 | Gingival crevicular fluid proteomes in health, gingivitis and chronic periodontitis. | Different study population |
| 54 | Jaedicke KM et al., 2016 | Salivary cytokines as biomarkers of periodontal diseases. | Review |
| 55 | Kainat R et al., 2023 | Assessment of Salivary MMP-8 and IL-1β for the Diagnosis of Periodontal Diseases in Pakistani Population | Different study population |
| 56 | Kasuma N et al., 2018 | The analysis of matrix metalloproteinase-8 in gingival crevicular fluid and periodontal diseases | Different study population |
| 57 | Katsiki P et al., 2021 | Comparing periodontitis biomarkers in saliva, oral rinse and gingival crevicular fluid: A pilot study | Different study population |
| 58 | Keskin M et al., 2023 | A Comparative Analysis of Treatment-Related Changes in the Diagnostic Biomarker Active Metalloproteinase-8 Levels in Patients with Periodontitis. Diagnostics | Different study population |
| 59 | Khan S et al., 2024 | Salivary tumour necrosis factor-alpha as a diagnostic marker of desquamative gingivitis. | Different study population |
| 60 | Khongkhunthian S et al., 2013 | Elevated levels of a disintegrin and metalloproteinase 8 in gingival crevicular fluid of patients with periodontal diseases. | Different study population |
| 61 | Kim HD et al., 2014 | Validation of periodontitis screening model using sociodemographic, systemic, and molecular information in a Korean population | Different study population |
| 62 | Kinney JS et al., 2014 | Crevicular fluid biomarkers and periodontal disease progression | Different study population |
| 63 | Konopka et al., 2012 | Gingival Crevicular Fluid MMP-8 Level in Patients with Chronic Periodontitis. | Different language |
| 64 | Konopka L et al., 2012 | Effect of scaling and root planing on interleukin-1β, interleukin-8 and MMP-8 levels in gingival crevicular fluid from chronic periodontitis patients. | Different aim/study group |
| 65 | Lamberts BL et al., 1989 | Fibronectin levels of unstimulated saliva from naval recruits with and without chronic inflammatory periodontal disease. | Different study population |
| 66 | Lee A et al., 2012 | Bacterial and salivary biomarkers predict the gingival inflammatory profile. | Different study population |
| 67 | Liukkonen J et al., 2016 | Salivary Concentrations of Interleukin (IL)-1β, IL-17A, and IL-23 Vary in Relation to Periodontal Status. | Different study population |
| 68 | Lundtorp-Olsen C et al., 2024 | Supragingival microbiota, cytokines, and proteins in individuals with different trajectories in experimental gingivitis | Different study population |
| 69 | Lundy FT et al., 2009 | Neuropeptide Y (NPY) and NPY Y1 receptor in periodontal health and disease | Different study population |
| 70 | Madruga D et al., 2023 | Positive correlational shift between crevicular antimicrobial peptide LL-37, pain and periodontal status following non-surgical periodontal therapy. A pilot study. | Different study population |
| 71 | Mäntylä P et al., 2003 | Gingival crevicular fluid collagenase-2 (MMP-8) test stick for chair-side monitoring of periodontitis | Different study population |
| 72 | Markkanen H et al., 1986 | Salivary IgA, lysozyme and beta 2-microglobulin in periodontal disease. | Different study population |
| 73 | Martinez GL et al., 2017 | Salivary Colony Stimulating Factor-1 and Interleukin-34 in Periodontal Disease | Different study population |
| 74 | Miller CS et al., 2006 | Salivary biomarkers of existing periodontal disease: a cross-sectional study**.** | Different study population |
| 75 | Nalmpantis D et al., 2020 | Azurocidin in gingival crevicular fluid as a potential biomarker of chronic periodontitis | Different study population |
| 76 | Parlak HM et ak., 2022 | Statherin and alpha-amylase levels in saliva from patients with gingivitis and periodontitis | Different study population |
| 77 | Rai B et al., 2008 | Biomarkers of periodontitis in oral fluids. | Different study population |
| 78 | Räisänen IT et al., 2019 | On the diagnostic discrimination ability of mouthrinse and salivary aMMP-8 point-of-care testing regarding periodontal health and disease. | Different aim |
| 79 | Räisänen IT et al., 2019 | A point-of-care test of active-matrix metalloproteinase-8 predicts triggering receptor expressed on myeloid cells-1 (TREM-1) levels in saliva. | Different aim |
| 80 | Räisänen IT et al., 2018 | Point-of-Care/Chairside aMMP-8 Analytics of Periodontal Diseases' Activity and Episodic Progression. Diagnostics (Basel). | Different aim |
| 81 | Räisänen IT et al., 2021 | Low association between bleeding on probing propensity and the salivary aMMP-8 levels in adolescents with gingivitis and stage I periodontitis. | Different aim |
| 82 | Räisänen IT et al., 2019 | Active-Matrix Metalloproteinase-8 Point-of-Care (PoC)/Chairside Mouthrinse Test vs. Bleeding on Probing in Diagnosing Subclinical Periodontitis in Adolescents | Different aim |
| 83 | Ramenzoni LL et al., 2021 | Origin of MMP-8 and Lactoferrin levels from gingival crevicular fluid, salivary glands and whole saliva | Different study population |
| 84 | Rathinasamy K et al., 2020 | Estimation of TNF-α Levels in Saliva and Serum of Patients with Periodontal Health and Chronic Periodontitis: A Case-control Study | Different study population |
| 85 | Ribeiro et al., 2020 | Salivary Expression of Antimicrobial Peptide LL37 and Its Correlation with Pro-inflammatory Cytokines in Patients with Different Periodontal Treatment Needs. | Different study population |
| 86 | Sağlam M et al., 2015 | Levels of interleukin-37 in gingival crevicular fluid, saliva, or plasma in periodontal disease. | Different study population |
| 87 | Schmidt J et al., 2018 | aMMP-8 in correlation to caries and periodontal condition in adolescents-results of the epidemiologic LIFE child study | Different aim |
| 88 | Shin MS et al., 2019 | Deep sequencing salivary proteins for periodontitis using proteomics. | Different study population |
| 89 | Van Dyke T et al., 2002 | Clinical and microbial evaluation of a histatin-containing mouthrinse in humans with experimental gingivitis: a phase-2 multi-center study | Different aim/study population |

REFERENCES:

1. Abdullameer MA, Abdulkareem AA**.** Salivary interleukin-1β as a biomarker to differentiate between periodontal health, gingivitis, and periodontitis. Minerva Dent Oral Sci. 2023 Oct., 72(5):221-229. doi: 10.23736/S2724-6329.23.04778-2. Epub 2023 May 10. PMID: 37162330**.**
2. Acquier AB, Pita AK, Busch L, Sánchez GA. Comparison of salivary levels of mucin and amylase and their relation with clinical parameters obtained from patients with aggressive and chronic periodontal disease. J Appl Oral Sci. 2015 May-Jun., 23(3):288-94. doi: 10.1590/1678-775720140458. PMID: 26221923., PMCID: PMC4510663
3. Afacan B, Öztürk VÖ, Emingil G, Köse T, Bostanci N. Alarm anti-protease trappin-2 negatively correlates with proinflammatory cytokines in patients with periodontitis. J Periodontol. 2018 Jan., 89(1):58-66. doi: 10.1902/jop.2017.170245. PMID: 28777039.
4. Afacan B, Öztürk VÖ, Paşalı Ç, Bozkurt E, Köse T, Emingil G. Gingival crevicular fluid and salivary HIF-1α, VEGF, and TNF-α levels in periodontal health and disease. J Periodontol. 2019 Jul., 90(7):788-797. doi: 10.1002/JPER.18-0412. Epub 2018 Dec 11. PMID: 30536725.
5. Aji NRAS, Räisänen IT, Rathnayake N, Lundy FT, Mc Crudden MTC, Goyal L, Sorsa T, Gupta S. aMMP-8 POCT vs. Other Potential Biomarkers in Chair-Side Diagnostics and Treatment Monitoring of Severe Periodontitis. Int J Mol Sci. 2024 Aug 30., 25(17):9421. doi: 10.3390/ijms25179421. PMID: 39273368., PMCID: PMC11395035.
6. Akhi R, Nissinen AE, Wang C, Kyrklund M, Paju S, Mäntylä P, Buhlin K, Sinisalo J, Pussinen PJ, Hörkkö S. Salivary IgA antibody to malondialdehyde-acetaldehyde associates with mild periodontal pocket depth. Oral Dis. 2022 Nov., 28(8):2285-2293. doi: 10.1111/odi.13936. Epub 2021 Jun 14. PMID: 34124817.
7. Alhammadi A, Koippallil Gopalakrishnan AR, Saqan R, Badran Z, Al Kawas S, Rahman B. Salivary macrophage chemokines as potential biomarkers of gingivitis. BMC Oral Health. 2023 Feb 6., 23(1):77. doi: 10.1186/s12903-023-02787-5. PMID: 36747174., PMCID: PMC9903476.
8. Armitage GC. Analysis of gingival crevice fluid and risk of progression of periodontitis. Periodontol 2000. 2004., 34:109-19. doi: 10.1046/j.0906-6713.2002.003427.x. PMID: 14717858.
9. Baeza M, Garrido M, Hernández-Ríos P, Dezerega A, García-Sesnich J, Strauss F, Aitken JP, Lesaffre E, Vanbelle S, Gamonal J, Brignardello-Petersen R, Tervahartiala T, Sorsa T, Hernández M. Diagnostic accuracy for apical and chronic periodontitis biomarkers in gingival crevicular fluid: an exploratory study. J Clin Periodontol. 2016 Jan., 43(1):34-45. doi: 10.1111/jcpe.12479. Epub 2016 Feb 12. PMID: 26556177.
10. Baliban RC, Sakellari D, Li Z, DiMaggio PA, Garcia BA, Floudas CA. Novel protein identification methods for biomarker discovery via a proteomic analysis of periodontally healthy and diseased gingival crevicular fluid samples. J Clin Periodontol. 2012 Mar., 39(3):203-12. doi: 10.1111/j.1600-051X.2011.01805.x. Epub 2011 Nov 10. PMID: 22092770., PMCID: PMC3268946.
11. Baron AC, Gansky SA, Ryder MI, Featherstone JD. Cysteine protease inhibitory activity and levels of salivary cystatins in whole saliva of periodontally diseased patients. J Periodontal Res. 1999 Nov., 34(8):437-44. doi: 10.1111/j.1600-0765.1999.tb02279.x. PMID: 10697800.
12. Belstrøm D, Damgaard C, Könönen E, Gürsoy M, Holmstrup P, Gürsoy UK. Salivary cytokine levels in early gingival inflammation. J Oral Microbiol. 2017 Aug 11., 9(1):1364101. doi: 10.1080/20002297.2017.1364101. PMID: 28839521., PMCID: PMC5560406.
13. Bildt MM, Bloemen M, Kuijpers-Jagtman AM, Von den Hoff JW. Collagenolytic fragments and active gelatinase complexes in periodontitis. J Periodontol. 2008 Sep., 79(9):1704-11. doi: 10.1902/jop.2008.080021. PMID: 18771372.
14. Bostanci N, Bao K. Contribution of proteomics to our understanding of periodontal inflammation. Proteomics. 2017 Feb., 17(3-4). doi: 10.1002/pmic.201500518. PMID: 27995754.
15. Buduneli N, Kinane DF. Host-derived diagnostic markers related to soft tissue destruction and bone degradation in periodontitis. J Clin Periodontol. 2011 Mar., 38 Suppl 11:85-105. doi: 10.1111/j.1600-051X.2010.01670.x. PMID: 21323706.
16. Caffesse RG, Quiñones CR. Polypeptide growth factors and attachment proteins in periodontal wound healing and regeneration. Periodontol 2000. 1993 Feb., 1(1):69-79. PMID: 8401862.
17. Castagnola M, Congiu D, Denotti G, Di Nunzio A, Fadda MB, Melis S, Messana I, Misiti F, Murtas R, Olianas A, Piras V, Pittau A, Puddu G. Determination of the human salivary peptides histatins 1, 3, 5 and statherin by high-performance liquid chromatography and by diode-array detection. J Chromatogr B Biomed Sci Appl. 2001 Feb 10., 751(1):153-60. doi: 10.1016/s0378-4347(00)00466-7. PMID: 11232845.
18. Chung WO, Dommisch H, Yin L, Dale BA. Expression of defensins in gingiva and their role in periodontal health and disease. Curr Pharm Des. 2007., 13(30):3073-83. doi: 10.2174/138161207782110435. PMID: 17979750.
19. Costa LCM, Soldati KR, Fonseca DC, Costa JE, Abreu MHNG, Costa FO, Zandim-Barcelos DL, Cota LOM. Gingival crevicular fluid levels of human beta-defensin 1 in individuals with and without chronic periodontitis. J Periodontal Res. 2018 Oct., 53(5):736-742. doi: 10.1111/jre.12558. Epub 2018 Apr 23. PMID: 29687452.
20. da Silva CVF, Bacila Sade Y, Naressi Scapin SM, da Silva-Boghossian CM, de Oliveira Santos E. Comparative proteomics of saliva of healthy and gingivitis individuals from Rio de Janeiro. Proteomics Clin Appl. 2023 Sep., 17(5):e2200098. doi: 10.1002/prca.202200098. Epub 2023 Feb 17. PMID: 36764829.
21. Dale BA, Kimball JR, Krisanaprakornkit S, Roberts F, Robinovitch M, O'Neal R, Valore EV, Ganz T, Anderson GM, Weinberg A. Localized antimicrobial peptide expression in human gingiva. J Periodontal Res. 2001 Oct., 36(5):285-94. doi: 10.1034/j.1600-0765.2001.360503.x. PMID: 11585115.
22. De Smet K, Contreras R. Human antimicrobial peptides: defensins, cathelicidins and histatins. Biotechnol Lett. 2005 Sep., 27(18):1337-47. doi: 10.1007/s10529-005-0936-5. PMID: 16215847.
23. de Souza-Gugelmin MC, Ito IY, Maia Campos G. Creation of the gingival immunologic defense index (GIDI) to evaluate the immunological potential of the gingiva and the possible risk for periodontal disease. Braz Dent J. 1995., 6(2):95-1-2. PMID: 8688664.
24. De-Gennaro LA, Lopes JD, Mariano M. Autoantibodies directed to extracellular matrix components in patients with different clinical forms of periodontitis. J Periodontol. 2006 Dec., 77(12):2025-30. doi: 10.1902/jop.2006.060104. PMID: 17209787.
25. Embery G, Waddington R. Gingival crevicular fluid: biomarkers of periodontal tissue activity. Adv Dent Res. 1994 Jul., 8(2):329-36. doi: 10.1177/08959374940080022901. PMID: 7865094.
26. Ersin Kalkan R, Öngöz Dede F, Gökmenoğlu C, Kara C. Salivary fetuin-A, S100A12, and high-sensitivity C-reactive protein levels in periodontal diseases. Oral Dis. 2018 Nov., 24(8):1554-1561. doi: 10.1111/odi.12927. Epub 2018 Jul 18. PMID: 29949223.
27. Fábián TK, Hermann P, Beck A, Fejérdy P, Fábián G. Salivary defense proteins: their network and role in innate and acquired oral immunity. Int J Mol Sci. 2012., 13(4):4295-4320. doi: 10.3390/ijms13044295. Epub 2012 Apr 2. PMID: 22605979., PMCID: PMC3344215.
28. Ford PJ, Gamonal J, Seymour GJ. Immunological differences and similarities between chronic periodontitis and aggressive periodontitis. Periodontol 2000. 2010 Jun., 53:111-23. doi: 10.1111/j.1600-0757.2010.00349.x. PMID: 20403108.
29. Front E, Laster Z, Unis R, Gavish M, Nagler RM. Salivary biomarker analysis complementing regular clinical examination. Biomark Med. 2013 Oct., 7(5):701-8. doi: 10.2217/bmm.13.76. PMID: 24044562.
30. Galunska, Bistra Tzaneva, Salim, Ayshe Seyhan, Nikolova, Miglena Nikolaeva, Angelova, Sirma Todorova, Kiselova-Kaneva, Yoana Dimitrova, Peev, Stefan Vasilev and Ivanova, Diana Georgieva. "Gingival status and prophylactic oral hygiene measures modulate salivary amino acids’ profile in children with plaque-induced gingivitis" *Turkish Journal of Biochemistry*, vol. 49, no. 1, 2024, pp. 47-55. [https://doi.org/10.1515/tjb-2023-01](https://doi.org/10.1515/tjb-2023-0107)
31. Gonçalves Lda R, Soares MR, Nogueira FC, Garcia C, Camisasca DR, Domont G, Feitosa AC, Pereira Dde A, Zingali RB, Alves G. Comparative proteomic analysis of whole saliva from chronic periodontitis patients. J Proteomics. 2010 May 7., 73(7):1334-41. doi: 10.1016/j.jprot.2010.02.018. Epub 2010 Mar 4. PMID: 20215060.
32. Gonçalves PF, Huang H, McAninley S, Alfant B, Harrison P, Aukhil I, Walker C, Shaddox LM. Periodontal treatment reduces matrix metalloproteinase levels in localized aggressive periodontitis. J Periodontol. 2013 Dec., 84(12):1801-8. doi: 10.1902/jop.2013.130002. Epub 2013 Mar 28. PMID: 23537121., PMCID: PMC4418528.
33. Görgülü NG, Doğan B. Effect of non-surgical periodontal treatment on salivary and serum biomarkers in Stage III Grade B and C periodontitis. J Periodontol. 2022 Aug., 93(8):1191-1205. doi: 10.1002/JPER.21-0536. Epub 2022 Feb 21. PMID: 35043972.
34. N. G. GÖRGÜLÜ And B. DOĞAN, "Salivary MMP-8, MAF, and IL-34 levels in non-surgical periodontal treatment of gingivitis patients," *Europerio 10* , vol.49, Denmark, pp.146-147, 2022
35. Groenink J, Walgreen-Weterings E, Nazmi K, Bolscher JG, Veerman EC, van Winkelhoff AJ, Nieuw Amerongen AV. Salivary lactoferrin and low-Mr mucin MG2 in Actinobacillus actinomycetemcomitans-associated periodontitis. J Clin Periodontol. 1999 May., 26(5):269-75. doi: 10.1034/j.1600-051x.1999.260501.x. PMID: 10355615.
36. Güncü GN, Yilmaz D, Könönen E, Gürsoy UK. Salivary Antimicrobial Peptides in Early Detection of Periodontitis. Front Cell Infect Microbiol. 2015 Dec 24., 5:99. doi: 10.3389/fcimb.2015.00099. PMID: 26734583., PMCID: PMC4689996.
37. Gupta S, Sahni V, Räisänen IT, Grigoriadis A, Sakellari D, Gieselmann DR, Sorsa T. Linking oral microbial proteolysis to aMMP-8 PoC diagnostics along with the stage and grade of periodontitis: A cross-sectional study. Oral Dis. 2023 Jan., 29(1):285-289. doi: 10.1111/odi.14008. Epub 2021 Aug 27. PMID: 34402146.
38. Gursoy UK, Liukkonen J, Jula A, Huumonen S, Suominen AL, Puukka P, Könönen E. Associations Between Salivary Bone Metabolism Markers and Periodontal Breakdown. J Periodontol. 2016 Apr., 87(4):367-75. doi: 10.1902/jop.2015.150399. Epub 2015 Nov 26. PMID: 26609698.
39. Güven O, De Visscher JG. Salivary IgA in periodontal disease. J Periodontol. 1982 May., 53(5):334-5. doi: 10.1902/jop.1982.53.5.334. PMID: 6953232.
40. Hadzic Z, Pasic E, Hukic M, Vukelic MG, Hadzic S. Salivary Interleukin-6 Levels in Patients with Periodontitis Stage IV. Meandros Med Dent J 2021., 22:140-147.
41. Hägewald SJ, Fishel DL, Christan CE, Bernimoulin JP, Kage A. Salivary IgA in response to periodontal treatment. Eur J Oral Sci. 2003 Jun., 111(3):203-8. doi: 10.1034/j.1600-0722.2003.00040.x. PMID: 12786950.
42. Hartenbach FARR, Velasquez É, Nogueira FCS, Domont GB, Ferreira E, Colombo APV. Proteomic analysis of whole saliva in chronic periodontitis. J Proteomics. 2020 Feb 20., 213:103602. doi: 10.1016/j.jprot.2019.103602. Epub 2019 Dec 4. PMID: 31809901
43. Hayakawa H, Yamashita K, Ohwaki K, Sawa M, Noguchi T, Iwata K, Hayakawa T. Collagenase activity and tissue inhibitor of metalloproteinases-1 (TIMP-1) content in human whole saliva from clinically healthy and periodontally diseased subjects. J Periodontal Res. 1994 Sep., 29(5):305-8. doi: 10.1111/j.1600-0765.1994.tb01226.x. PMID: 7799209.
44. Heikkinen AM, Raivisto T, Räisänen IT, Sorsa T. Prevention of Initial Periodontitis Is an Investment in the Future**.** Diagnostics (Basel). 2024 Aug 24., 14(17):1850. doi: 10.3390/diagnostics14171850. PMID: 39272635., PMCID: PMC11394008.
45. Heikkinen AM, Nwhator SO, Rathnayake N, Mäntylä P, Vatanen P, Sorsa T. Pilot Study on Oral Health Status as Assessed by an Active Matrix Metalloproteinase-8 Chairside Mouthrinse Test in Adolescents. J Periodontol. 2016 Jan., 87(1):36-40. doi: 10.1902/jop.2015.150377. Epub 2015 Oct 2. PMID: 26430926
46. Heikkinen AM, Raivisto T, Räisänen I, Tervahartiala T, Bostanci N, Sorsa T. Implementing of aMMP-8 point-of-care test with a modified new disease classification in Finnish adolescent cohorts. Clin Exp Dent Res. 2022 Oct., 8(5):1142-1148. doi: 10.1002/cre2.603. Epub 2022 Jun 8. PMID: 35676762., PMCID: PMC9562567.
47. Henskens YM, Van der Velden U, Veerman EC, Nieuw Amerongen AV. Cystatin C levels of whole saliva are increased in periodontal patients. Ann N Y Acad Sci. 1993 Sep 20., 694:280-2. doi: 10.1111/j.1749-6632.1993.tb18364.x. PMID: 8215066.
48. Henskens YM, van der Velden U, Veerman EC, Nieuw Amerongen AV. Protein, albumin and cystatin concentrations in saliva of healthy subjects and of patients with gingivitis or periodontitis. J Periodontal Res. 1993 Jan., 28(1):43-8. doi: 10.1111/j.1600-0765.1993.tb01049.x. PMID: 8426281.
49. Henskens, Y. M. C., van der Weijden, G. A., Veerman, E. C. I., & van Nieuw Amerongen, A. (1995). Salivary cystatins and their relation to periodontal disease. Journal of Dental Research, 74, 503.
50. Hernández M, Baeza M, Räisänen IT, Contreras J, Tervahartiala T, Chaparro A, Sorsa T, Hernández-Ríos P. Active MMP-8 Quantitative Test as an Adjunctive Tool for Early Diagnosis of Periodontitis. Diagnostics (Basel). 2021 Aug 20., 11(8):1503. doi: 10.3390/diagnostics11081503. PMID: 34441437., PMCID: PMC8394100.
51. Hong I, Pae HC, Song YW, Cha JK, Lee JS, Paik JW, Choi SH. Oral Fluid Biomarkers for Diagnosing Gingivitis in Human: A Cross-Sectional Study. J Clin Med. 2020 Jun 3., 9(6):1720. doi: 10.3390/jcm9061720. PMID: 32503210., PMCID: PMC7356847.
52. Hormia M, Thesleff I, Perheentupa J, Pesonen K, Saxén L. Increased rate of salivary epidermal growth factor secretion in patients with juvenile periodontitis. Scand J Dent Res. 1993 Jun., 101(3):138-44. doi: 10.1111/j.1600-0722.1993.tb01653.x. PMID: 8322007.
53. Huynh AH, Veith PD, McGregor NR, Adams GG, Chen D, Reynolds EC, Ngo LH, Darby IB. Gingival crevicular fluid proteomes in health, gingivitis and chronic periodontitis. J Periodontal Res. 2015 Oct., 50(5):637-49. doi: 10.1111/jre.12244. Epub 2014 Nov 29. PMID: 25439677.
54. Jaedicke KM, Preshaw PM, Taylor JJ. Salivary cytokines as biomarkers of periodontal diseases. Periodontol 2000. 2016 Feb., 70(1):164-83. doi: 10.1111/prd.12117. PMID: 26662489.
55. Kainat R, Ahmed I, Alolaywi AM, Waheed H, Sultan ZK, Moin SF. Assessment of Salivary MMP-8 and IL-1β for the Diagnosis of Periodontal Diseases in Pakistani Population. Eur J Dent. 2024 May., 18(2):672-679. doi: 10.1055/s-0043-1772779. Epub 2023 Dec 12. PMID: 38086426., PMCID: PMC11132764.
56. Kasuma N, Oenzil F, Darwin E, Sofyan Y. The analysis of matrix metalloproteinase-8 in gingival crevicular fluid and periodontal diseases. Indian J Dent Res. 2018 Jul-Aug., 29(4):450-454. doi: 10.4103/ijdr.IJDR_97_15. PMID: 30127195.
57. Katsiki P, Nazmi K, Loos BG, Laine ML, Schaap K, Hepdenizli E, Bikker FJ, Brand HS, Veerman ECI, Nicu EA. Comparing periodontitis biomarkers in saliva, oral rinse and gingival crevicular fluid: A pilot study. J Clin Periodontol. 2021 Sep., 48(9):1250-1259. doi: 10.1111/jcpe.13479. Epub 2021 Jun 20. PMID: 33998029., PMCID: PMC8453974.
58. Keskin M, Rintamarttunen J, Gülçiçek E, Räisänen IT, Gupta S, Tervahartiala T, Pätilä T, Sorsa T. A Comparative Analysis of Treatment-Related Changes in the Diagnostic Biomarker Active Metalloproteinase-8 Levels in Patients with Periodontitis. Diagnostics (Basel). 2023 Feb 27., 13(5):903. doi: 10.3390/diagnostics13050903. PMID: 36900047., PMCID: PMC10001139.
59. Khan S, Bey A, Bansal P, Moin S, Rahman SZ. Salivary tumour necrosis factor-alpha as a diagnostic marker of desquamative gingivitis. Bangladesh Journal of Medical Science. 2024 Mar 27., 23(2):484-90.
60. Khongkhunthian S, Techasatian P, Supanchart C, Bandhaya P, Montreekachon P, Thawanaphong S, Krisanaprakornkit S. Elevated levels of a disintegrin and metalloproteinase 8 in gingival crevicular fluid of patients with periodontal diseases. J Periodontol. 2013 Apr., 84(4):520-8. doi: 10.1902/jop.2012.120262. Epub 2012 May 21. PMID: 22612366.
61. Kim HD, Sukhbaatar M, Shin M, Ahn YB, Yoo WS. Validation of periodontitis screening model using sociodemographic, systemic, and molecular information in a Korean population. J Periodontol. 2014 Dec., 85(12):1676-83. doi: 10.1902/jop.2014.140061. PMID: 24965062.
62. Kinney JS, Morelli T, Oh M, Braun TM, Ramseier CA, Sugai JV, Giannobile WV. Crevicular fluid biomarkers and periodontal disease progression. J Clin Periodontol. 2014 Feb., 41(2):113-120. doi: 10.1111/jcpe.12194. Epub 2013 Dec 12. PMID: 24303954., PMCID: PMC4247885.
63. Konopka, Łukasz and Ewa Brzezińska‐Błaszczyk. “Gingival Crevicular Fluid MMP-8 Level in Patients with Chronic Periodontitis.” Dental and Medical Problems 49 (2012): 543-549.
64. Konopka L, Pietrzak A, Brzezińska-Błaszczyk E. Effect of scaling and root planing on interleukin-1β, interleukin-8 and MMP-8 levels in gingival crevicular fluid from chronic periodontitis patients. J Periodontal Res. 2012 Dec., 47(6):681-8. doi: 10.1111/j.1600-0765.2012.01480.x. Epub 2012 Apr 18. PMID: 22510045.
65. Lamberts BL, Pederson ED, Bial JJ, Tombasco PK. Fibronectin levels of unstimulated saliva from naval recruits with and without chronic inflammatory periodontal disease. J Clin Periodontol. 1989 Jul., 16(6):342-6. doi: 10.1111/j.1600-051x.1989.tb00002.x. PMID: 2668347.
66. Lee A, Ghaname CB, Braun TM, Sugai JV, Teles RP, Loesche WJ, Kornman KS, Giannobile WV, Kinney JS. Bacterial and salivary biomarkers predict the gingival inflammatory profile. J Periodontol. 2012 Jan., 83(1):79-89. doi: 10.1902/jop.2011.110060. Epub 2011 May 12. PMID: 21563952.
67. Liukkonen J, Gürsoy UK, Pussinen PJ, Suominen AL, Könönen E. Salivary Concentrations of Interleukin (IL)-1β, IL-17A, and IL-23 Vary in Relation to Periodontal Status. J Periodontol. 2016 Dec., 87(12):1484-1491. doi: 10.1902/jop.2016.160146. Epub 2016 Aug 19. PMID: 27541079.
68. Lundtorp-Olsen C, Nygaard N, Massarenti L, Constancias F, Damgaard C, Kahraman Gursoy U, van Splunter A, Bikker FJ, Gursoy M, Markvart M, Belstrøm D. Supragingival microbiota, cytokines, and proteins in individuals with different trajectories in experimental gingivitis. J Oral Microbiol. 2024 Jul 5., 16(1):2372861. doi: 10.1080/20002297.2024.2372861. PMID: 38979478., PMCID: PMC11229773.
69. Lundy FT, El Karim IA, Linden GJ. Neuropeptide Y (NPY) and NPY Y1 receptor in periodontal health and disease. Arch Oral Biol. 2009 Mar., 54(3):258-62. doi: 10.1016/j.archoralbio.2008.10.002. Epub 2008 Nov 17. PMID: 19010457.
70. Madruga D, Garcia MM, Martino L, Hassan H, Elayat G, Ghali L, Ceballos L. Positive correlational shift between crevicular antimicrobial peptide LL-37, pain and periodontal status following non-surgical periodontal therapy. A pilot study. BMC Oral Health. 2023 May 28., 23(1):335. doi: 10.1186/s12903-023-03023-w. PMID: 37246231., PMCID: PMC10226254.
71. Mäntylä P, Stenman M, Kinane DF, Tikanoja S, Luoto H, Salo T, Sorsa T. Gingival crevicular fluid collagenase-2 (MMP-8) test stick for chair-side monitoring of periodontitis. J Periodontal Res. 2003 Aug., 38(4):436-9. doi: 10.1034/j.1600-0765.2003.00677.x. PMID: 12828663.
72. Markkanen H, Syrjänen SM, Alakuijala P. Salivary IgA, lysozyme and beta 2-microglobulin in periodontal disease. Scand J Dent Res. 1986 Apr., 94(2):115-20. doi: 10.1111/j.1600-0722.1986.tb01374.x. PMID: 3518036.
73. Martinez GL, Majster M, Bjurshammar N, Johannsen A, Figueredo CM, Boström EA. Salivary Colony Stimulating Factor-1 and Interleukin-34 in Periodontal Disease. J Periodontol. 2017 Aug., 88(8):e140-e149. doi: 10.1902/jop.2017.170081. Epub 2017 May 5. PMID: 28474967.
74. Miller CS, King CP Jr, Langub MC, Kryscio RJ, Thomas MV. Salivary biomarkers of existing periodontal disease: a cross-sectional study. J Am Dent Assoc. 2006 Mar., 137(3):322-9. doi: 10.14219/jada.archive.2006.0181. PMID: 16570465.
75. Nalmpantis D, Gatou A, Fragkioudakis I, Margariti A, Skoura L, Sakellari D. Azurocidin in gingival crevicular fluid as a potential biomarker of chronic periodontitis. J Periodontal Res. 2020 Apr., 55(2):209-214. doi: 10.1111/jre.12703. Epub 2019 Oct 14. PMID: 31608993.
76. Parlak HM, Buber E, Gur AT, Karabulut E, Akalin FA. Statherin and alpha-amylase levels in saliva from patients with gingivitis and periodontitis. Arch Oral Biol. 2023 Jan., 145:105574. doi: 10.1016/j.archoralbio.2022.105574. Epub 2022 Oct 22. PMID: 36395562.
77. Rai B, Kharb S, Jain R, Anand SC. Biomarkers of periodontitis in oral fluids. J Oral Sci. 2008 Mar., 50(1):53-6. doi: 10.2334/josnusd.50.53. PMID: 18403884.
78. Räisänen IT, Heikkinen AM, Nwhator SO, Umeizudike KA, Tervahartiala T, Sorsa T. On the diagnostic discrimination ability of mouthrinse and salivary aMMP-8 point-of-care testing regarding periodontal health and disease. Diagn Microbiol Infect Dis. 2019 Dec., 95(4):114871. doi: 10.1016/j.diagmicrobio.2019.114871. Epub 2019 Jul 26. PMID: 31473032.
79. Räisänen IT, Heikkinen AM, Pakbaznejad Esmaeili E, Tervahartiala T, Pajukanta R, Silbereisen A, Bostanci N, Sorsa T. A point-of-care test of active matrix metalloproteinase-8 predicts triggering receptor expressed on myeloid cells-1 (TREM-1) levels in saliva. J Periodontol. 2020 Jan., 91(1):102-109. doi: 10.1002/JPER.19-0132. Epub 2019 Aug 12. PMID: 31343739
80. Räisänen IT, Heikkinen AM, Siren E, Tervahartiala T, Gieselmann DR, van der Schoor GJ, van der Schoor P, Sorsa T**.** Point-of-Care/Chairside aMMP-8 Analytics of Periodontal Diseases' Activity and Episodic Progression. Diagnostics (Basel). 2018 Oct 22., 8(4):74. doi: 10.3390/diagnostics8040074. PMID: 30360358., PMCID: PMC6315514.
81. Räisänen IT, Sorsa T, Tervahartiala T, Raivisto T, Heikkinen AM. Low association between bleeding on probing propensity and the salivary aMMP-8 levels in adolescents with gingivitis and stage I periodontitis. J Periodontal Res. 2021 Apr., 56(2):289-297. doi: 10.1111/jre.12817. Epub 2020 Dec 11. PMID: 33305834.
82. Räisänen IT, Sorsa T, van der Schoor GJ, Tervahartiala T, van der Schoor P, Gieselmann DR, Heikkinen AM. Active-Matrix Metalloproteinase-8 Point-of-Care (PoC)/Chairside Mouthrinse Test vs. Bleeding on Probing in Diagnosing Subclinical Periodontitis in Adolescents**.** Diagnostics (Basel). 2019 Mar 23., 9(1):34. doi: 10.3390/diagnostics9010034. PMID: 30909530., PMCID: PMC6468891.(Different aim comparing two index tests)
83. Ramenzoni LL, Hofer D, Solderer A, Wiedemeier D, Attin T, Schmidlin PR. Origin of MMP-8 and Lactoferrin levels from gingival crevicular fluid, salivary glands and whole saliva. BMC Oral Health. 2021 Aug 5., 21(1):385. doi: 10.1186/s12903-021-01743-5. PMID: 34353321., PMCID: PMC8340507.
84. Rathinasamy K, Ulaganathan A, Ramamurthy S, Ganesan R, Saket P, Alamelu S. Estimation of TNF-α Levels in Saliva and Serum of Patients with Periodontal Health and Chronic Periodontitis: A Case-control Study. J Contemp Dent Pract. 2020 Feb 1., 21(2):148-151. PMID: 32381818.
85. Ribeiro, Ana Elisa Rodrigues Alves et al. “Salivary Expression of Antimicrobial Peptide LL37 and Its Correlation with Pro-inflammatory Cytokines in Patients with Different Periodontal Treatment Needs.” International Journal of Peptide Research and Therapeutics 26 (2020): 2547-2553.
86. Sağlam M, Köseoğlu S, Savran L, Pekbağriyanik T, Sağlam G, Sütçü R. Levels of interleukin-37 in gingival crevicular fluid, saliva, or plasma in periodontal disease. J Periodontal Res. 2015 Oct., 50(5):614-21. doi: 10.1111/jre.12241. Epub 2014 Nov 17. PMID: 25399716.
87. Schmidt J, Guder U, Kreuz M, Löffler M, Kiess W, Hirsch C, Ziebolz D, Haak R. aMMP-8 in correlation to caries and periodontal condition in adolescents-results of the epidemiologic LIFE child study. Clin Oral Investig. 2018 Jan., 22(1):449-460. doi: 10.1007/s00784-017-2132-0. Epub 2017 Jun 4. PMID: 28578462.
88. Shin MS, Kim YG, Shin YJ, Ko BJ, Kim S, Kim HD. Deep sequencing salivary proteins for periodontitis using proteomics. Clin Oral Investig. 2019 Sep., 23(9):3571-3580. doi: 10.1007/s00784-018-2779-1. Epub 2018 Dec 15. PMID: 30554327.
89. Van Dyke T, Paquette D, Grossi S, Braman V, Massaro J, D'Agostino R, Dibart S, Friden P. Clinical and microbial evaluation of a histatin-containing mouthrinse in humans with experimental gingivitis: a phase-2 multi-center study. J Clin Periodontol. 2002 Feb., 29(2):168-76. doi: 10.1034/j.1600-051x.2002.290212.x. PMID: 11895545
